# Warmth and competence predict overoptimistic beliefs for out-group but not in-group members

**DOI:** 10.1371/journal.pone.0207670

**Published:** 2018-11-26

**Authors:** Mihai Dricu, Stephanie Bührer, Fabienne Hesse, Cecily Eder, Andres Posada, Tatjana Aue

**Affiliations:** 1 Department of Experimental Psychology and Neuropsychology, University of Bern, Bern, Switzerland; 2 Department of Applied Psychology: Health, Development, Enhancement and Intervention, University of Vienna, Vienna, Austria; 3 Faculty of Psychology and Educational Sciences, University of Geneva, Geneva, Switzerland; University of Rome, ITALY

## Abstract

People can be overly optimistic not only about their own future but also for the people with whom they identify. Furthermore, interpersonal perception generally forms along two universal dimensions, i.e. warmth and competence. In this study, we created four fictional characters that would map onto each quadrant of the two-dimensional space of warmth and competence, i.e. one in-group member (high on both warmth and competence) and three out-group members (high warmth, low competence; high competence, low warmth; low on both warmth and competence). We then asked respondents to assess the likelihood of each character experiencing a series of identical desirable and undesirable events in order to uncover potential optimistic biases. Our study had two goals. First, we wanted to balance the target desirable and undesirable events on four key characteristics, i.e. event frequency, controllability, emotional intensity and personal experience with the event. Second, we wanted to investigate whether stereotypes of warmth and competence could influence the respondents’ likelihood estimates for each character. We show that respondents manifested a strong desirability bias, expecting more desirable than undesirable events for the in-group member and the reverse pattern for the extreme out-group member. More important, we show that, within desirable and undesirable events, respondents anchored their judgments for the in-group member on their personal experience with the target events, further revealing an egocentric bias, but turned to stereotypical knowledge in the form of warmth and competence to judge out-group members. Implications for both social perception and optimism research are discussed.

## Introduction

Probabilities are a fundamental aspect of experience. Events ranging from the large and consequential (‘‘Will this surgical procedure be successful?”) to the mundane (‘‘Will the train be on time?”) are all characterized by a lack of certainty, falling along a continuum of likelihood. An event’s perceived likelihood is then critical in influencing the choices we make regarding the event. For instance, we are more likely to participate in a medical procedure if we believe to have a high rather than low chance of being cured.

Often there can be a discrepancy between what people expect will happen in general and what they think is likely to happen to them personally [1, 2]. Research has consistently shown that people are optimistically biased in producing their own likelihoods of experiencing events, i.e. positive outcomes are expected to occur more often and negative outcomes to occur less often [3–16]. Such systematic optimistic bias is known by many names in the research literature [13]. Sheppard, Klein, Waters, & Weinstein [14] asked for consistency in labelling this phenomenon as *unrealistic optimism* and reviewed two main types of unrealistic optimism: absolute and comparative. Unrealistic absolute optimism refers to a personal estimate for a future event that is optimistically inaccurate as compared to an objective standard, e.g. overestimating one’s likelihood of winning the lottery in comparison with the actual statistical odds of winning. Unrealistic comparative optimism also involves a personal likelihood that is favorable, but the comparison is made to the likelihood for a reference person, e.g. assessing one’s own likelihood of winning the lottery as greater than a colleague’s likelihood of winning. The current study focuses on unrealistic comparative optimism. While absolute optimism results solely from biased personal likelihoods, comparative optimism may result from a distortion in personal likelihoods of future events, a distortion in the perceived likelihood of the comparison target, or both.

### Moderators of optimistic biases

Optimistic biases are particularly resistant to outside influence, for instance when tempted with large incentives for making accurate estimations [17, 18]. Nevertheless, researchers have found a series of factors that can moderate the magnitude and the direction of overoptimistic beliefs, such as the characteristics of the events in question [15, 19–22] and characteristics of the reference target [11, 23–30]. These moderators are discussed in more detail in the following sections.

One question that arises is whether simultaneously balancing different event characteristics influences the emergence of the optimistic bias. Past studies have predominantly investigated one event characteristic at a time (e.g. [31–34]). The first goal of our study was therefore balancing desirable and undesirable future events on key event characteristics, namely perceived frequency, controllability, emotional impact and personal experience with the event. As such, half of our events were low, and half were high on each of these four characteristics. Furthermore, by using such a diverse palette of future events, one can properly investigate which event characteristics, if any, influence overoptimistic beliefs. Past studies have found conflicting results with regard to the patterns of influence of event characteristics on likelihood estimates [20, 30, 34–36]. One likely reason for such contrary evidence is the fact these studies have not simultaneously balanced the target events on other key characteristics, thus potentially leading to misleading results.

Another question that emerges is whether individuals estimate the likelihoods of events differently for different reference targets, above and beyond influences from event characteristics. Independent lines of research on the optimistic bias have investigated likelihood estimates for oneself and the average other [21, 37], and for in-group and out-group members [18, 38]. The second goal of our study was to provide better conceptualization of the reference target in the form of a known social psychological model [39] and investigate how the optimistic bias manifests with regard to different social groups.

The following two sections will describe the two sets of moderators of the optimistic bias, i.e. event characteristics and reference target characteristics. We will then describe the social psychological model used to improve the conceptualization and predictive power of the reference target. We will finally bring all this information together and state our hypotheses in the last section of the introduction.

#### Event characteristics

The most scrutinized event characteristics that moderate overoptimistic beliefs have been the target event’s perceived controllability [21, 32, 33], the event’s desirability [40–42], the event’s frequency [14, 20], and prior experience or exposure to the event in question [5, 43–46]. The perceived controllability of an event refers to the perception that one has the capability, resources or opportunities to maximize positive outcomes and to avoid negative consequences through one’s own actions [47], or collective actions such as community-based interventions [48, 49] and institutional programs [50]. Early reviews and meta-analyses showed a positive relationship between perceived event controllability and optimistic bias for undesirable events, such that the greater the perceived control over the occurrence or the outcome of an event, the greater the optimistic bias for that event (i.e. the less likely it is expected to happen [21, 37]). More recent correlational studies [33, 44, 51] and regression analyses [20, 25, 28, 45] further support the notion that people make similar judgments for themselves and others about the likelihoods of uncontrollable events but showcase optimistic beliefs about controllable events. Interestingly, Hoorens, Smits, & Sheppard [52] took another approach, i.e. instead of providing ready-made scenarios, they let participants freely generate potential future events and subsequently asked them to rate the events on valence and controllability, as well as on the chances of occurrence for themselves and for others. The authors found no differences between estimates for oneself and others for uncontrollable events but found optimistic beliefs for controllable events, such that more desirable events and fewer undesirable events were generated for oneself than for others.

There is compelling evidence that an event’s perceived frequency also has a considerable influence on whether optimistic beliefs are formed, with rare events more strongly triggering comparative optimistic beliefs than common events [15, 20, 25, 27, 53]. One of the reasons for this phenomenon is the way we process self-relevant information [20]. As such, the high frequency of an event makes it more salient in one’s mind, leading to the rapid assessment of its high occurrence. However, a rare event necessitates more active memory search and retrieval, more guesswork, thus exposing the mind of the respondent to the optimistic bias. Interestingly, participants tend to rate the perceived frequency of events by extrapolating their own experience and prior exposure to the events in question[15, 54]. In other words, the higher the exposure to an event, the higher the perceived frequency of it. Consequently, some studies have focused instead on the role of experience rather than perceived frequency in explaining the mechanisms of overoptimistic beliefs. What these studies have found is that optimistic bias is stronger when past experience with undesirable events (either firsthand or second-hand) is insufficient or scarce [11, 32, 37, 44–46, 55–57], thus mirroring the findings on perceived event frequency.

Additionally, an event’s perceived desirability is hypothesized to play an important role when judging the chances of experiencing them [31, 41, 42, 58]. When asked to imagine their chances of experiencing an event in the future, respondents might experience affective reactions towards the target situation, such as the event’s inherent valence (e.g. how pleasurable an event would be) or the consequences of the event (e.g. an event’s severity). These affective reactions then create motivational tendencies to approach desirable events and avoid undesirable events, which, in turn, increase likelihood estimates for desirable future events and lower estimates for undesirable future events [42, 59].

Although optimistic bias is often defined in relation to both undesirable and desirable events [60–62], much of our understanding of the mechanisms that underlie optimistic beliefs comes from studies on undesirable events and outcomes, probably because of their more important consequences for health prevention and intervention [12]. Researchers generally assume that moderators of optimistic beliefs operate in parallel directions depending on the desirability of the events, such that people believe that desirable events will happen more often to them and that undesirable events will happen less often compared to others [61, 63]. However, this assumption has often been challenged, with some studies showing that overoptimistic beliefs for desirable events are weaker compared to undesirable events [35, 64] or even absent [20, 30]. One likely reason for this inconsistency is that desirable and undesirable events have not been closely matched on other characteristics, e.g. perceived event frequency, controllability or emotional intensity [65, 66]. In addition to this *biased sampling of events*, several other methodological concerns have been voiced, such as *scale attenuation* and *minority underrepresentation* [12, 20, 67, 68]. Scale attenuation refers to the type of response format that participants generally use to give their answers. It has been argued that a narrow scale (e.g. a typical seven point Likert scale from “much less likely = -3” to “much more likely = +3”) would inflate the magnitude of overoptimistic beliefs by not considering more nuanced differences in likelihoods[12, 67]. Minority underrepresentation refers to the consequence of sampling too many rare events combined with a non-representative sampling of respondents, which would lead to an overall disproportionate number of respondents who will not experience the target events compared to those who might experience them. Consequently, the overall pool of respondents might seem biased towards overoptimistic, when in fact, they could be accurate in their predictions.

In summary, several event characteristics can exert a considerable influence on the magnitude of optimistic beliefs. However, because of consistent methodological confounds, including the biased sampling of events characteristics, the true prevalence of the optimistic bias has been questioned [12, 66]. The present study will address these concerns by balancing target events of key characteristics and investigate the extent to which overoptimistic beliefs still manifest. Furthermore, this diverse palette of future events will allow us to properly investigate which event characteristics influence overoptimistic beliefs.

#### Reference target characteristics

Because unrealistic comparative optimism requires a reference target, extensive research has focused on the nature of the reference target and how it might impact the optimistic bias. The most studied characteristics of the reference target have been the *level of specificity* [11, 23, 27, 30] and the *degree of closeness* between the self and the reference target [1, 11, 24, 26, 28, 29, 37, 51, 69].

In the typical experiment on optimistic bias, participants are asked to compare themselves with the average other, usually of the same age and gender. It has been argued that such comparison is too abstract and that it allows people considerable leeway in the selection of the comparison target, who then tend to choose targets that are stereotypical and convenient for the comparison at hand in order to maintain optimistic beliefs, which, in turn, serve psychological well-being [11]. By asking people to compare themselves against a concrete person (e.g. someone known), this liberty is restricted, and the optimistic bias is reduced [11, 23, 26, 27, 30]. Recent research has taken this approach one step further and has focused on the degree of closeness between the self and the comparison target, revealing that the optimistic bias can be removed altogether if the target is someone close to the respondents [37]. In other words, we are optimistic not only about ourselves but also about the people close to us. Despite converging results, *closeness* has been conceptualized inconsistently, either as social distance [24], vocational similarity [1], self-generated similarity [28], the amount of information known about someone [69] or left entirely to the understanding of the participant (e.g. “your best friend” [11, 26, 29, 30, 51]). As a result, no consistent pattern of underlying mechanisms has been offered for these results, other than the parsimonious account of wishful thinking—that respondents believe what they wish to believe, and that they provide likelihood estimates accordingly [41, 58]. People care about their close ones more than they care about other people and are thus motivated in wishing to see them experiencing more desirable events and less undesirable events. Having desires affects the way that people search for and process information, and the optimistic conclusions that they reach are the result of this biased information processing [41, 70].

In an independent line of research, sports fans also display optimistic beliefs about the likelihood of their favorite team winning the game [17, 38, 71–74]. Similarly, voters display optimistic beliefs about the chances of their preferred political candidate winning elections [18, 75–78], with the opposing candidates expected to lose by a significant margin [18, 76]. Researchers have found a direct relationship between the amount of overoptimistic beliefs that sports fans and voters display, on one hand, and the direction and magnitude of identification with the sports team or political candidates, on the other hand [72, 75]. While these findings are not directly relevant to the ones on unrealistic comparative optimism (i.e. emerging as a comparison between oneself and a relevant other), they can be understood as an extension to forming optimistic beliefs. We are overoptimistic not only about our own likelihoods and of those close to us but also about the likelihoods of those we identify with, all the while being pessimistic about the likelihoods of those we identify less with.

The overoptimistic beliefs of sports fans and political voters are motivated by in-group favoritism and out-group discrimination [72, 76], which, in turn, are motivated by a need to reach and maintain positive self-esteem [79–83]. By evaluating groups and other individuals that we identify with more favorably than others on a given attribute (e.g. chances of experiencing certain events), we are actively maintaining our positive self-esteem via group membership and a distinctive social identity [79, 81, 84, 85]. Because closeness, similarity and social distance are outcomes of social identification processes [86, 87], we argue that the same motivational processes that underlie optimistic beliefs about favorite sports teams and preferred political candidates (i.e. maintaining self-esteem via social identification processes) can be extended to traditionally studied comparison target characteristics (i.e. closeness, similarity). In other words, we expect that the mechanisms which lead to and maintain in-group favoritism and out-group discrimination can explain the results of overoptimistic beliefs in various life domains, whether the reference target is someone close to us or an unknown other, a favorite sports team or a preferred political candidate.

Research has focused not only on *why* in-group favoritism and out-group discrimination appear, but also on *when* and *how*. In-group favoritism and out-group discrimination, through which we maintain a positive social identity [79], are not omnipresent but instead manifest only in certain domains of life, depending on the status and power relations of the in-group in relation to other groups [88–93]. Specifically, in order to maximize the positivity of one’s social identity, the dimension that is most favorable for the in-group and least favorable for the out-group will be used for the social comparison [92, 94–99]. The domains of social comparison in which groups pursue positively distinct identities map on two broad dimensions that have been variously described as agency, autonomy or competence, on one hand, and warmth, expressiveness and communion, on the other [39, 88, 95–98]. The Stereotype Content Model predicts how the dimensions of competence and warmth dictate emotions, attitudes and behaviors between groups [89]. For example, Oldmeadow and Fiske [98] found that members of a prestigious university choose to differentiate themselves positively on stereotypes of competence (e.g. intelligence, performance), whereas members of a less prestigious university differentiate themselves positively on stereotypes of warmth (e.g. social skills, trustworthiness, sociability). Because hypotheses in the present research were substantially influenced by the Stereotype Content Model, we will describe it in more detail in the next section.

### Stereotype content model

According to the Stereotype Content Model (SCM), all social groups and their members are perceived as possessing a unique combination of *warmth* and *competence* mapped on a two-dimensional space [39, 90, 100]. At the same time, all groups are aware of their perceived position in this space [88, 101–103]. Depending on their interaction goals, groups may either emphasize the dimensions on which they are perceived as being strong (e.g. high-status individuals may promote their own personal competence when interacting with other high-status individuals) or minimize their standing on their stereotyped dimension (e.g. high-status individuals may downplay their stereotypical status and promote themselves as everyday people when interacting with low-status individuals; [96, 97, 104, 105]).

The warmth dimension is analogous to perceived communal qualities such as friendliness, trustworthiness, empathy and kindness [100, 101, 106, 107], whereas the competence dimension conveys perceived agentic virtues such as intelligence, skill, and power [39, 90]. In simple terms, stereotypes of warmth elicit emotions and behaviors akin to like-dislike, while stereotypes of competence elicit emotions and behaviors akin to respect-disrespect [106, 107]. The two-dimensional space of warmth-competence then results in four sets of stereotypes: two univalent stereotypes (one high on both warmth and competence; one low on both warmth and competence) and two mixed stereotypes (high on one dimension and low on the other [39, 108]).

Each of the four stereotypes prescribes its own set of emotions, attitudes and behaviors [39, 89, 91, 92, 103, 109]. Groups perceived as high on both competence and warmth trigger pride and admiration, attitudes of like and respect, and behaviors of active help and passive facilitation. Such groups are prevalent, social reference groups (e.g. the middle class, Caucasians in the Western World). Groups perceived as low on both competence and warmth trigger disgust and contempt, attitudes of dislike and disrespect, and behaviors of active harm (harassment, bullying) and passive harm (e.g. neglect, exclusion). Such groups frequently include welfare beneficiaries, and substance abusers[89]. Mixed groups perceived as high on warmth but low on competence trigger pity, attitudes of like but disrespect, and behaviors of passive harm (e.g. neglect, exclusion) combined with active facilitation (e.g. assisting, defending). In Western communities, such groups consistently include housewives and the elderly [94, 109]. Mixed groups perceived as high on competence but low on warmth trigger envy and resentment, attitudes of dislike but respect, and behaviors of active harm (e.g. harassment, belittling) and passive facilitation (e.g. associating with, networking). Successful businesspeople are often included in this mixed group [39, 90, 110].

In the previous section, we argued that overoptimistic beliefs are motivated by in-group favoritism and out-group discrimination [72, 76], which, in turn, stem from selective intra-group evaluations on dimensions of warmth and competence [89, 91]. It should follow, therefore, that overoptimistic beliefs can appear as the result of the emotional and cognitive intra-group evaluations emerging from stereotypes of warmth and competence. Correspondingly, in the present study, we investigated the extent to which the dimensions of warmth and competence may influence the estimate likelihoods of the reference target experiencing a series of preselected events.

### Current study

The present study had two aims. First, we wanted to address some of the methodological concerns surrounding research on comparative optimism [12, 66]. To overcome biased sampling of events, our study balanced four event characteristics across desirable and undesirable events, namely perceived event frequency, event controllability, emotional intensity of the events and the personal experience with the events. As shown above, these event characteristics can moderate the magnitude and direction of overoptimistic beliefs [21, 37]. If left unbalanced, either of these characteristics might confound results and bias their interpretation. Furthermore, by using a percentage response scale from 0% to 100%, we allowed our participants to choose more nuanced responses that would otherwise not be possible with a typical attenuated Likert scale [66]. Second, we wanted to better specify the reference target in terms of warmth and competence, and to investigate how these dimensions would influence the reference target’s perceived likelihood estimates for desirable and undesirable events. Stereotypes of warmth and competence incorporate previously investigated characteristics of the reference target such as closeness and similarity [88, 90, 100, 111] but they provide a clearer conceptualization and higher predictive power [89, 108].

To reach these aims, we took several approaches. We first conceived of thirty-two events (sixteen desirable and sixteen undesirable) that would match on controllability (high and low) and frequency (high and low). Because emotional intensity and personal experience refer to event characteristics that are subjectively appraised by the respondents, such data could only be collected after the events had been generated. A manipulation check of whether the events are balanced on personal experience and emotional intensity could therefore only be achieved post-hoc. In a second step, we created four animated characters as representatives of social groups that have been previously mapped on each quadrant of the warmth-competence two-dimensional space [39, 89, 90, 92, 103, 109, 110, 112, 113]. Because we sampled student participants, we chose a student character with whom participants would implicitly identify as their de facto in-group (i.e. implicitly perceived as high on warmth and high on competence [24, 79, 80, 82, 84]). In addition to the student character, three other characters were created as stereotypical representatives of out-groups: an alcoholic character (low on perceived warmth and competence), a businessperson (high on perceived competence but low on warmth) and an elderly person (high on perceived warmth but low on competence). These three characters and their associated pervasive stereotypes have been previously validated in numerous cross-national samples from Europe, Americas, Asia, Oceania and Africa [94, 109, 112]. We then asked the student participants to assess the likelihoods of each character experiencing the thirty-two events in a randomized fashion. We expected that implicit intergroup evaluations on stereotypes of warmth and competence would overall drive the respective likelihood estimates (i.e. chances of experiencing an event).

Across characters, and within desirable and undesirable events, we expected participants to display comparative optimism for some characters relative to others, as dictated by the SCM. Because the competence dimension is defined by stereotypes of intelligence, efficiency, skill, and confidence [88, 90, 114], we expected that competent characters will be perceived as more capable of maximizing positive gains and avoiding negative outcomes. In turn, less competent characters will not be as capable of exerting any meaningful influence over events [88, 94, 109]. Therefore, we hypothesized that:

**Hypothesis 1a**: Participants will expect that competent characters would overall experience more desirable events than less competent characters.

**Hypothesis 1b**: Participants will expect that competent characters would overall experience less undesirable events than less competent characters.

Above and beyond competence levels, the warmth dimension is expected to play an indirect role in likelihood estimates. Specifically, warm characters are actively helped and supported by others in pursuing desirable situations and activities, and avoiding undesirable situations, whereas cold characters are actively hindered by others [102, 106, 109].

**Hypothesis 2a**: Participants will expect that the warm characters would overall experience more desirable events than cold characters.

**Hypothesis 2b**: Participants will expect that the warm characters would overall experience less undesirable events than cold characters.

Consequently, participants will expect that the competent and warm character would experience desirable events most often whereas the cold and less competent character would experience desirable events least often. Similarly, participants will expect that the competent and warm character would experience undesirable events least often whereas the cold and less competent character would experience desirable events most often. Members of mixed stereotypes (high on one dimension but low on the other) would be rated intermediately between the two univalent groups for desirable and undesirable events.

Furthermore, we wanted to investigate which of the event characteristics, if any, predict the likelihoods estimates for the four characters. Several studies have found conflicting results with regard to the patterns of influence of event characteristics, such as frequency [34, 36] and valence [20, 30, 35]. One likely reason for such discrepancy is the fact these studies have not simultaneously balanced the target events on other key characteristics. Therefore, we cannot make any strong a priori hypotheses about the direction of the influence, if any, of event characteristics on any of the likelihood estimates.

## Methods

### Participants

Eighty-Nine Swiss university students participated in this study. They were recruited via e-mails, fliers, and the local participant pool at the University of Bern. In exchange for their participation, they received either course credits or monetary payment. As inclusion criteria, participants had to be German speaking university students and aged between 18 and 30 years (M = 22.16, SD = 2.36). The sample consisted of 80 females (89.89%) and 9 males (10.11%). All participants gave informed and written consent for their participation. The local ethics committee of the University of Bern approved all experimental protocols and methods of data collection, data handling and data analysis. All methods and experimental protocols were performed in accordance with the guidelines and regulations of the local ethics committee of the University of Bern.

### Data collection

#### The events

Optimistic beliefs can only be formed in relation to potential future events. To address concerns over methodological and statistical confounds [12, 66], our aim was to choose a sample of events that were balanced on valence, frequency and controllability in the general population. To our knowledge, only one other study performed by Chambers, Windschitl & Suls attempted to balance these characteristics [20]. We argue, however, that the authors did not successfully reach their goal on two counts. First, Chambers et al. [20] did not publish the scores on perceived frequency, controllability and valence obtained in a pre-test study, and did not report any manipulation checks to determine whether the event characteristics were indeed balanced. Second, the pre-test ratings on these three attributes were obtained with an unusual procedure that lends itself to high inter-subject variability. Specifically, the authors used a low sample size of raters per event classification in terms of desirability (i.e., valence), frequency, and controllability (“Pretest participants (N = 57) were randomly assigned to rate a subset (approximately one third) of a total of 128 events”, p.1350) and a separate pool of participants assessed the likelihood estimates of the chosen events (N = 63, main experiment). In our study, by contrast, we asked the same sample of participants to rate the same events on perceived valence, emotional intensity, frequency, controllability, and personal experience, and to assess likelihood estimates for four reference targets.

A pool of events balanced on valence (desirable and undesirable), frequency (frequent and rare) and controllability (high and low) would result in eight possible combinations. To sample as many situations as possible while keeping the experiment length at a minimum, we generated four events for each of the eight combinations. We selected twenty-two events out of the original sample from Chambers et al. [20] and we supplemented them with eleven other events, of which seven were newly created and four were rephrased and adapted from Chambers et al. [20]. The final sample of the thirty-two events are listed in **S2 Table**.

#### The task

A second aim of our study was to better address the previously studied comparison target characteristics of closeness and similarity and re-conceptualize them as stemming from the universal dimensions of warmth and competence. As such, the experimental task required participants to give likelihood estimates for four fictional characters experiencing each of the thirty-two events.

These four characters were chosen to reflect each of the quadrants of the two-dimensional space of warmth and competence, as predicted by the SCM model[89, 91]. A student character served as the implicit in-group for our participants, high on both warmth and competence [24, 79, 80, 82]. Three characters were created to serve as relevant out-groups. A univalent out-group (low on both warmth and competence) consistently found across cultures and samples is formed by welfare recipients and substance users [89]. As such, we created a character that was alcoholic. Two characters further served as prototypical members of mixed out-groups: an elderly person (high on warmth but low on competence; [109]) and a successful businessperson (high on competence but low on warmth; [39, 90, 110]).

**Fig 1:**
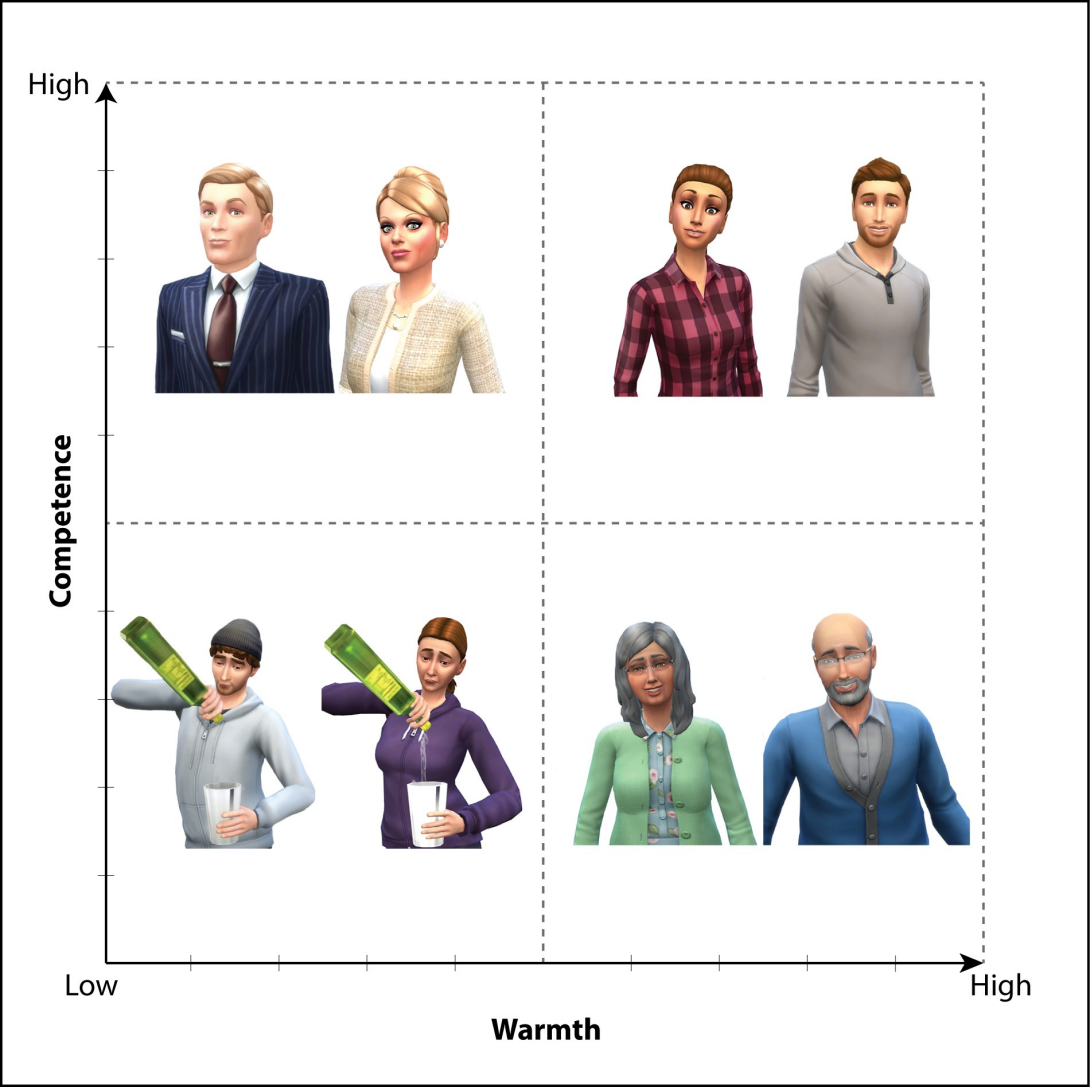
The four characters based on the stereotype content model. A male and a female version of each character was created and each participants only viewed same-gender versions of the characters.

To ease the participants’ task, we created still animations to illustrate each character in each scenario. Four standalone animated characters reflecting the SCM stereotypes were first created to accompany task instructions (**Fig 1**). Additionally, one hundred and twenty-eight situations were created to reflect each character in each of the thirty-two events discussed above, with one set of events per character. Separately, thirty-two backgrounds pertinent to the target events were created onto which the four sets of events were carefully cropped by aligning size and position. All characters, backgrounds and scenarios were created using *The Sims 4* (Electronic Arts, California, USA). The final one hundred and twenty-eight stimuli depicting the four characters in thirty-two events (**S4 Fig**) were matched in brightness and contrast using *Matlab R2017a* (The MathWorks, Inc. Massachusetts, USA). To avoid possible gender influences, for every stereotype and scenario we created a male and a female version. Female participants saw female animated characters while male participants viewed male animated characters.

#### Experimental procedure

Data collection took place at the University of Bern, Switzerland. Forty-one participants were tested in a behavioral testing room that could simultaneously accommodate up to five individuals. Forty-eight participants additionally underwent a physiological acquisition procedure and were recruited individually. Only the behavioral data is presented and discussed in the current manuscript. All participants first received a short booklet with explanations regarding the alleged nature of the study along with the written informed consent, which had been previously approved by the local ethics committee and in accordance with the Declaration of Helsinki. The communicated purpose of the study was piloting a series of stimuli for a future experiment. After signing the consent form, the participants could start the experiment.

The experiment was programmed with E-Prime 2.0 Professional (version 2.0.10.353; Psychology Software Tools, Pittsburgh, USA) and consisted of six parts, each preceded by written instructions displayed on the computer screen. The first part asked participants to familiarize themselves with the four characters and informed them of the specific task, i.e. providing likelihood estimates for each of the four characters experiencing each of the thirty-two scenarios. A total of one hundred and twenty-eight trials were displayed in a randomized order and without interruptions. Each trial started with a jittered fixation cross (1.5s-3s), followed by a one-sentence description of the target event displayed in the middle of the screen for 2s, and subsequently followed by a 10s screen that displayed the target scenario (i.e. a single still animation of the target character involved in the target event) during which the participants could choose a likelihood score with a continuous visual analog scale from 0% to 100% (**S5 Fig**). The experiment was self-paced so that once a likelihood estimate was chosen, the participants could press Enter to immediately continue to the next trial. If no selection was made, the choice “50” was automatically registered for that event.

The second part of the experiment consisted of instructions displayed on the screen that asked participants to provide *personal* likelihood estimates for experiencing the same thirty-two events. A total of thirty-two trials were displayed in a randomized order and without interruptions. Each trial started with a jittered fixation cross (1.5s-3s), followed by a one-sentence description of the target event displayed for 2s, and subsequently followed by a 10s screen with the word “Yourself” displayed along with the same continuous visual analog scale from 0% to 100% that asked the participants to provide the likelihood score (**S6 Fig**). No other visual guidance and no still animations were provided. Like part one, the participants could press Enter to immediately continue to the next trial after choosing the preferred likelihood estimate. If no selection was made within the 10s window, the choice”50%” was automatically registered for that event.

The third part of the experiment asked participants for self-reported ratings of cognitive and emotional empathy for each of the four characters in each of the thirty-two scenarios. Due to space limitations, however, the data from this experimental part will not be presented here. The fourth part of the experiment consisted of target questions about the perceived event controllability, valence, and frequency as well as the emotional reaction and personal experience with the target event. As such, thirty-two trials were displayed in a randomized order and without interruptions. Each trial started with a jittered fixation cross (1.5s-3s), followed by a 10s screen containing a written one-sentence of the target scenario displayed in the center, and one of the five target questions (i.e. event controllability, event valence, event frequency, emotional intensity, personal experience) was displayed under the question. Participants could make their response with a continuous visual analog scale, displayed on the screen. The event controllability question was framed between “not at all controllable” and “very controllable”. The event frequency question was framed between “very rare” and “very frequent”. The question about the event valence was framed between “very negative” and “very positive”. The emotional intensity question was framed between “not at all emotional” and “very emotional”. Finally, the question about personal experience with the event was framed between “absolutely no experience” and “a lot of experience”. All scales had a clear demarcation in the middle without any label. To provide common units across the different scales and to ease subsequent analyses, the most leftward value of the scales was always 0, the most rightward value was always 100, and the middle value was 50. Any values between the left and right borders were registered as numbers with two decimals on a continuous scale between 0 and 100. We note that these values were chosen for convenience in subsequent statistical analyses and may not intuitively fit all scales. For example, scores of emotional valence could range from 0 (i.e. absolute negative valence) to 100 (i.e. absolute positive valence) with 50 in the middle (i.e. an emotionally neutral event) but common sense would imply that negative values are present in the left side of the scale and positive values in the right side of the scale. Nevertheless, our chosen values pose no difficulties for the interpretation of the scales.

The fifth part of the experiment asked participants about the perceived level of warmth and competence for each of the four characters. We included this part as a quick manipulation check of the SCM task. As such, four trials were displayed in a randomized order and without interruptions. During each trial, two consecutive windows were presented that assessed the level of warmth and the level of competence, respectively, with the help of a continuous visual analog scale. A still animation of the target character was displayed in the center of the screen, with the analog scale beneath. The warmth question was framed between “not at all warm” and “very warm”. The competence question was framed between “not at all competent” and “very competent”. This part did not have a time limit and was completely self-paced. The participants could continue to the next trial after making their choice and pressing Enter. We note that our participants were German speakers and the warmth dimension was evaluated using the German adjective “warmherzig” instead of “warm”. The most appropriate understanding of “warmherzig” in English is warmth in the social context, e.g. caring, affectionate, warm-hearted. Unlike English, the single German term “warm” more often refers to the physical property of temperature than the social domain, e.g. a warm blanket.

Finally, the sixth part of the experiment consisted of a single question assessing the Inclusion of Other in the Self (IOS; [115]). The IOS scale consists of a single pictorial item comprised of seven pairs of diagram-like overlapping circles on a continuum from a lesser to a greater overlap (S7 Fig). The degree of overlap depicted by each of the individual pairs represented a degree of interconnectedness (on a scale from one to seven). For each of the four characters, individuals were asked to choose the overlapping circles that best described their relationship with the character presented to the right side of the picture. We included the IOS scale as a manipulation check for whether participants identified with the student character as their implicit in-group more than with the out-group characters.

## Data analysis and results

### Data cleaning

We first proceeded with cleaning the data. Because our visual analog scale had the likelihood estimate of 50% as the default value and because the participants could move to the next trial by simply pressing enter, we wanted to identify the number of trials with this default value. On average, 8.09% of the trials consisted of the 50% default value (SD = 10.37%, ranging from 0% to 69%).

Because the default 50% value could still reflect genuine assessments of likelihood estimates, we wanted to flag instead those participants who were consistently choosing the default value, thus not paying sufficient attention to the task at hand. In addition, we wanted to flag extreme thinkers–those participants who consistently chose either the 0% likelihood estimate (i.e. absolutely no chances that this event would ever happen to the target character) or the 100% estimate likelihoods (i.e. absolute certainty that the target event will happen to the target character). To identify these participants, we calculated the percentages that the likelihood estimates of 0%, 50% and 100% received out of the total estimates at the level of each participant and then at the sample level (i.e. averaged across participants). At sample level, we additionally calculated the standard deviations of the percentages that the likelihood estimates of 0%, 50% and 100% received. Next, we flagged those participants whose percentages of either 0%, 50% or 100% likelihood estimates were more than three standard deviations above the percentages of estimates at the sample level.

Following this procedure, we identified seven outliers: two participants repeatedly chose the default scale value of 50% (38% and 69% of their total answers, respectively), one participant chose the 0% likelihood estimates in 16% of the trials, three participants frequently chose the 100% likelihood estimate (35%, 36% and 42% of their total answers, respectively) and one participant repeatedly chose both likelihood estimates of 0% (24% of all answers) and 100% (40% of all answers). All analyses reported below were conducted with the remaining sample of eighty-two participants. We note that running the analyses with and without the remaining trials with the values of 0%, 50% and 100% (i.e. after excluding the seven outliers) led to identical findings. Therefore, the analyses reported below were conducted including these trials.

The manipulation check of the event and the characters can be found in the Supplementary Material (S1 Analysis, S2 Analysis and S1 Discussion).

### Optimistic beliefs

#### The effects of warmth and competence on likelihood estimates

The main scope of our study was to determine whether the designated warmth and competence of the reference target would influence the latter’s likelihood estimates for desirable and undesirable events. In this regard, we conducted a three-way repeated measures ANOVA on the likelihood estimates, with designated warmth (high versus low), designated competence (high versus low) and desirability (desirable versus undesirable events) as our within-subject factors. There was a statistically significant three-way interaction between our factors (F (1,81) = 165.59, p < .0005, η_p_^2^ = .672), which we resolved with two-way ANOVAs separately for desirable and undesirable events.

**Fig 2:**
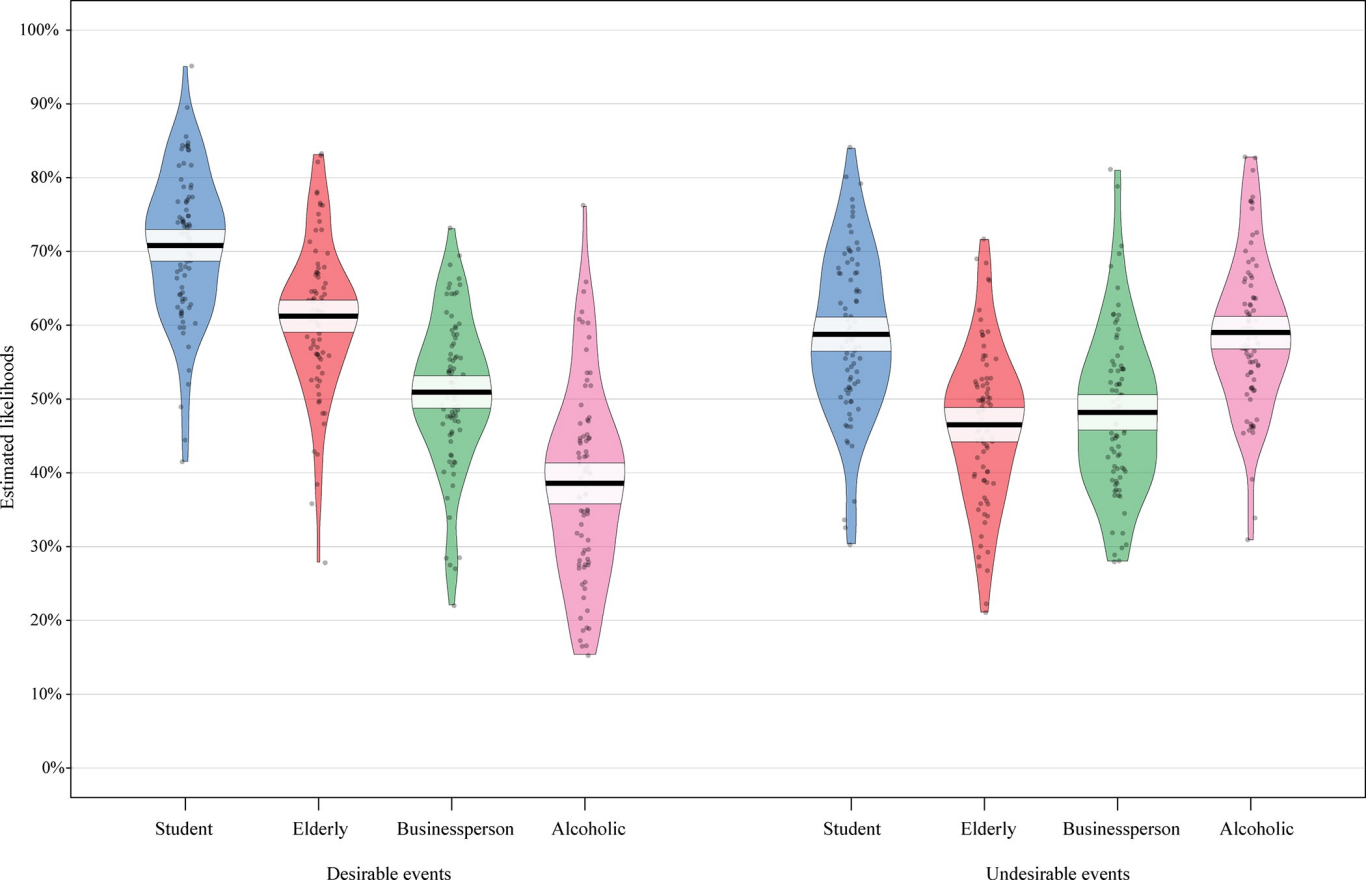
Pirate plots of the likelihood estimates that each character received for desirable and undesirable events. Dark horizontal lines reflect the sample mean. Horizontal bands around the sample mean reflect 95% confidence interval.

For desirable events, we found a main effect of competence (F (1,81) = 117.22, p < .0005, η_p_^2^ = .591), with competent characters (*M* = 60.87%, SD = 10.08%) expected to experience more desirable events than less competent characters (*M* = 49.92%, SD = 11.58%; **Fig 2**). This supports our hypothesis **H1a**. There was also a main effect of warmth (F (1,81) = 347.43, p < .0005, η_p_^2^ = .811), with warm characters (*M* = 66.02%, SD = 10.13%) expected to live more desirable events than cold characters (*M* = 44.77%, SD = 11.52%; **Fig 2**), thus supporting our hypothesis **H2a.** No statistically significant two-way interaction effect was found (F (1,81) = 2.48, p = .119, η_p_^2^ = .030).

For undesirable events, there was a significant two-way interaction between warmth and competence (F (1,81) = 185.32, p < .0005, η_p_^2^ = .696) but no significant main effects of warmth (F (1,88) = 1.98, p = .163, η_p_^2^ = .024) or competence (F (1,81) = .63, p = .429, η_p_^2^ = .008; **Fig 2**). Looking at the simple effects revealed that the participants expected competent cold characters (M = 48.17%, SD = 11.08%) to experience less undesirable events than less competent cold characters (M = 59.01%, SD = 10.38%, F (1,81) = 79.1030 p < .0005, η_p_^2^ = .495), but expected competent warm characters (M = 58.78%, SD = 10.99%) to experience more undesirable events than less competent warm characters (M = 46.51%, SD = 10.58%, F (1,81) = 96.59, p < .0005, η_p_^2^ = .544; **Fig 2**). Overall, our hypotheses **H1b** and **H2b** were not supported. Specifically, the respondents did not expect competent characters to experience overall less undesirable than less competent characters (i.e. lack of main effect of competence) and did not expect warm characters to experience overall less undesirable events than cold characters (i.e. lack of main effect of warmth).

**Fig 3:**
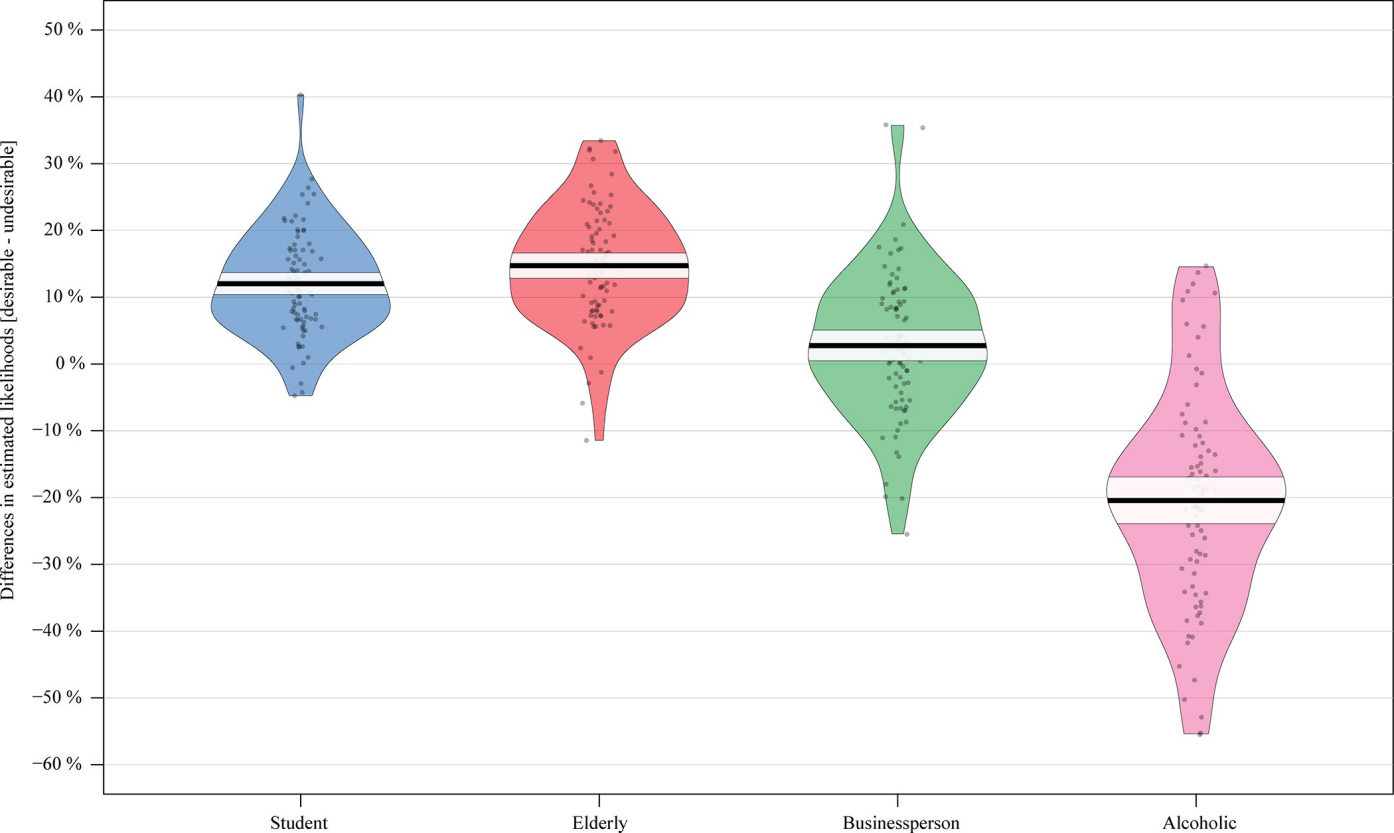
Pirate plots of the differences in likelihood estimates between desirable and undesirable events for each character. Positive values mean that desirable events are more likely than undesirable events. Dark horizontal lines reflect the sample mean. Horizontal bands around the sample mean reflect 95% confidence interval.

To better illustrate the simultaneous effects of warmth and competence on scores of desirable and undesirable events, we computed the difference in likelihood estimates between all averaged desirable events and all averaged undesirable events. This difference was computed at the level of each participant, separately for each of the four fictional characters and the self. Positive difference scores meant that, overall, desirable events were expected to be more likely than undesirable events. The opposite meaning was given to negative difference scores. We first conducted four one-sample t tests to reveal whether the differences for each character were significantly different from zero (alpha level = 0.013 Bonferroni correction for multiple testing). We found significant positive differences for the student character (t (81) = 13.78, p < .0005) and the elderly character (t (81) = 15.16, p < .0005) but a significant negative difference for the alcoholic character (t (81) = -11.32, p < .0005). The difference between desirable and undesirable events for the businessperson was not statistically significant from zero (t (81) = 2.29, p = .024). We then conducted a two-way repeated measures ANOVA with the factors warmth and competence on these difference scores. We found main effects of warmth (F (1,81) = 278.26, p < .0005, η_p_^2^ = .762) and competence (F (1,81) = 60.99, p < .0005, η_p_^2^ = .412), both being qualified by an interaction effect (F (1,81) = 130.99, p < .0005, η_p_^2^ = .601). A close look at simple effects revealed that the warm and less competent character (elderly; *M* = 14.50, MSE = .99, SD = 9.39) had a bigger difference in scores between desirable and undesirable events compared to the warm and competent character (student; *M* = 11.84, MSE = .95, SD = 8.96) but the effect size was small (F (1,81) = 5.14, p = .026, η_p_^2^ = .056; **Fig 3**). The cold and less competent character (alcoholic; *M* = -20.69, SD = 17.27) had a bigger difference in scores between desirable and undesirable events compared to the cold and less competent character (i.e. businessperson; *M* = 3.53, SD = 12.61) but in the opposite direction (F (1,81) = 111.54, p < .0005, η_p_^2^ = .562; **Fig 3**).

In summary, the analyses presented above converge on the finding that the warmth- and competence-informed hypotheses are supported for the three out-group members, but not the in-group member. To determine which factors might predict the likelihood estimates of the student character, we conducted a series of multiple regressions of the key event characteristics on the likelihood estimates of each character. While we did not have strong hypotheses about the direction of these results, the additional analyses are standard in comparative optimism research (e.g. [15, 20]) as they determine which of the event characteristics, if any, drive the likelihood estimates. We present these results in the following paragraphs.

#### Predicting likelihood estimates from event characteristics

In line with previous research on comparative optimism [15, 20, 24, 45, 66], we performed an additional set of analyses by considering the events (and not the participants) as the unit of analysis. To determine whether the participants judged the likelihood estimates from gauging one or more event characteristics, we first conducted a series of bivariate correlations between the five event characteristics and the five sets of likelihood estimates (**Table 1**). To compare the results on social optimism bias with those related to personal optimism bias, the estimates for oneself experiencing the target events were also considered.

**Table 1:** Bivariate correlations between the five event characteristics and the likelihood estimates.

Undesirable events	Self	Student	Elderly	Businessperson	Alcoholic
Valence	.227	.036	.276	.290	-.143
Intensity	-.243	-.282	-.018	.003	-.434
Controllability	.365	.301	.283	.563 ^a^	-.502 ^a^
Frequency	.942 ^a^	.884 ^a^	.725 ^a^	.310	.315
Experience	.986 ^a^	.887 ^a^	.770 ^a^	.318	.377
Desirable events	Self	Student	Elderly	Businessperson	Alcoholic
Valence	.424	.416	.078	-.231	-.165
Intensity	-.426	-.459	-.067	.406	-.058
Controllability	.345	.296	-.336	-.055	.611 ^a^
Frequency	.745 ^a^	.624 ^a^	-.072	.196	.409
Experience	.893 ^a^	.859 ^a^	.086	-.024	.211

*Note*. The table presents Pearson correlation coefficients.

^a^ p < .05, for all other entire p > .05

For desirable events, ratings of perceived event frequency significantly correlated with the likelihood estimates for the self (*r* = .94, p < .0005), the student character (*r* = .88, p < .0005) and the elderly character (*r* = .73, p = .001). Similarly, scores of personal experience with the events were significantly associated with the likelihood estimates for the self (*r* = .99, p < .0005), the student character (*r* = .89, p < .0005) and the elderly character (*r* = .77, p < .0005). Only the perceived controllability of an event was significantly associated with the likelihoods for the businessperson (*r* = .563, p = .023) and the alcoholic character (*r* = -.502, p = .049).

For undesirable events, ratings of event frequency significantly correlated with likelihood estimates for the self (*r* = .75, p = 0.001) and the student character (*r* = .62, p = 0.010), as did ratings of personal experience (self: *r* = .89, p < .0005; student: *r* = .86, p < .0005). Only the perceived controllability of an event was significantly associated with the likelihood estimates for alcoholic character (*r* = .61, p = .012). **Table 1** lists all Pearson correlation coefficients and their respective significance levels.

To determine which of the event characteristics would best predict the likelihood estimates for each fictional character and the self, we ran a series of ten standard multiple regressions controlling for repeated testing (adjusted alpha level of 0.005 using Bonferroni corrections; separate regressions for desirable and undesirable situations). Because emotional intensity was strongly associated with event valence, and because event frequency was highly correlated with personal experience, we only chose three event characteristics to be included as predictors in the regression models: personal experience with the event, perceived controllability and emotional intensity.

For desirable events, the multiple regression model with personal experience, event controllability and emotional intensity was statistically significant in predicting likelihood estimates for the self (F (3,12) = 139.83, p < .0005, adjusted R^2^ = .97) and the student character (F (3,12) = 15.04, p < .0005, adjusted R^2^ = .737) and the alcoholic character (F (3,12) = 7.59, p = .004, R^2^ = .568) but not the elderly character (F (3,12) = 6.69, p = .007, R^2^ = .532) or the businessperson (F (3,12) = 2.00, p = .168, adjusted R^2^ = .167). Likelihood estimates for the self were significantly predicted by scores of personal experience (p < .0005) but not event controllability (p = .666) or emotional intensity of events (p = .957). Similarly, likelihood estimates for the student were significantly predicted by personal experience (p < .0005) but not event controllability (p = .995) or emotional intensity (p = .666). Finally, likelihood estimates for the alcoholic character were significantly predicted by event controllability (p = .002) but not by the personal experience of the participants with the events (p = .013) or the emotional intensity of events (p = .190). Regression coefficients and standard errors for the desirable events can be found in **Table 2**.

**Table 2:** Regression coefficients and significance levels for the multiple regression models for desirable events.

Regression table for undesirable events.						
Dependent variable	Predictor	B	SE B	β	t	Sig.
Estimated likelihoods for self ^a^	(Constant)	20.070	8.312		2.415	0.033
	Personal experience with the event	.792	.042	.983	18.727	< .0005
	Event controllability	.006	.046	.007	.137	.666
	Emotional intensity of event	-.007	.118	-.003	-.063	.995
Estimated likelihoods for the student character ^b^	(Constant)	52.763	15.385		3.429	.005
	Personal experience with the event	.481	.082	.871	5.888	< .0005
	Event controllability	.001	.088	.001	.006	.995
	Emotional intensity of event	-.097	.220	-.061	-.443	.666
Estimated likelihoods for the alcoholic character ^c^	(Constant)	57.540	12.750		4.513	.001
	Personal experience with the event	.196	.068	.551	2.905	.013
	Event controllability	-.268	.073	-.677	-3.682	.002
	Emotional intensity of event	-.253	.182	-.247	-1.389	.190
Estimated likelihoods for the elderly character ^d^	(Constant)	14.356	24.331		.590	.566
	Personal experience with the event	.519	.124	.825	4.178	.001
	Event controllability	-.013	.134	-.019	-.098	.924
	Emotional intensity of event	.347	.335	.192	1.036	.351
Estimated likelihoods for the businessperson ^e^	(Constant)	31.684	27.774		1.141	.275
	Personal experience with the event	.078	.147	.139	.528	.608
	Event controllability	.319	.158	.514	2.012	.067
	Emotional intensity of event	.003	.397	.002	.008	.993

Note.

^a^ R^2^ = .97 (p < .0005)

^b^ R^2^ = .74 (p < .0005)

^c^ R^2^ = .57 (p = .004)

^d^ R^2^ = .53 (p = .007)

^e^ R^2^ = 17 (p = .168).

For undesirable events, the multiple regression model with personal experience, event controllability and emotional intensity was statistically significant in predicting likelihood estimates for the self (F (3,12) = 32.33, p < .0005, adjusted R^2^ = .86) and the student character (F (3,12) = 16.73, p < .0005, adjusted R^2^ = .76) but not the elderly character (F(3,12) = 1.14, p = .374, adjusted R^2^ = .03), the businessperson (F (3,12) = 1.71, p = .218, adjusted R^2^ = .13) or the alcoholic character (F (3,12) = 3.18, p = .063, adjusted R^2^ = .30; **Table 3**). Likelihood estimates for self were significantly predicted by scores of personal experience (p < .0005) but not event controllability (p = .227) or emotional intensity of the events (p = .020). Similarly, likelihood estimates for the student character were significantly predicted by scores of personal experience (p < .0005) but not event controllability (p = .206) or emotional intensity of the events (p = .201). Regression coefficients and standard errors can be found in **Table 3**.

**Table 3:** Regression coefficients and significance levels for the multiple regression models for undesirable events.

Dependent variable	Predictor	B	SE B	β	t	Sig.
Estimated likelihoods for self ^a^	(Constant)	1.631	.9778		.167	.870
	Personal experience with the event	.896	.104	1.226	8.621**	< .0005
	Event controllability	-0.095	.075	-0.147	-1.273	.227
	Emotional intensity of event	.282	.106	.362	2.668	.020
Estimated likelihoods for the student character ^b^	(Constant)	24.821	12.496		1.986	.070
	Personal experience with the event	.804	.133	1.139	6.051**	< .0005
	Event controllability	-.129	.096	-0.207	-1.352	.201
	Emotional intensity of event	.181	.135	.241	1.338	.206
Estimated likelihoods for the alcoholic character ^c^	(Constant)	25.410	19.772		1.285	.223
	Personal experience with the event	.018	.210	.028	.088	.932
	Event controllability	.434	.151	.745	2.872*	.014
	Emotional intensity of event	.220	.214	.315	1.031	.323
Estimated likelihoods for the elderly character ^d^	(Constant)	58.481	22.480		2.601	.023
	Personal experience with the event	.193	.239	.305	.806	.436
	Event controllability	-0.312	.172	-.557	-1.815	.095
	Emotional intensity of event	-0.079	.243	-0.118	-0.326	.750
Estimated likelihoods for the businessperson^e^	(Constant)	4.577	21.318		.215	.834
	Personal experience with the event	.311	.227	.492	1.371	.196
	Event controllability	.025	.163	.045	.155	.880
	Emotional intensity of event	.519	.230	.772	2.253	.044

Note.

^a^ R^2^ = .86 (p < .0005)

^b^ R^2^ = .76 (p < .0005)

^c^ R^2^ = .03 (p = .374)

^d^ R^2^ = .30 (p = .063)

^e^ R^2^ = 13 (p = .218).

## Discussion

Past literature has shown that people are overly optimistic about the chances of experiencing future events not only for themselves but also for people close to them and for those with whom they identify [21, 37]. However, these findings have been recently questioned on grounds of methodological confounds such as biased sampling of events and scale attenuation [12, 66]. In the present study, we addressed these confounds by balancing desirable and undesirable target events on several key characteristics, i.e. perceived frequency, controllability, emotional intensity and personal experience, and by including a response scale that allows for more nuanced answers, i.e. likelihood estimates from 0% to 100%. We additionally specified the reference target in terms of *warmth* and *competence*, two universal dimensions of social perception [88, 100, 108]. These dimensions incorporate previously studied characteristics of the reference target such as closeness and similarity but provide a clearer conceptualization and higher predictive power [89, 91, 103]. We created four fictional characters that would map onto each quadrant of the two-dimensional space of warmth and competence: a student character (univalent in-group member: high on warmth, high on competence), an alcoholic character (univalent out-group member: low on warmth, low on competence), an elderly character (mixed out-group member: high on warmth but low on competence), and a businessperson (mixed out-group member: high on competence but low on warmth). We then asked respondents to estimate the chances of each character experiencing identical target events. The main purpose of the study was to investigate how the stereotypes of warmth and competence elicited by the four characters would influence their likelihood estimates relative to each other. As predicted by the SCM, we hypothesized that respondents would show signs of comparative optimism for certain characters in relation to others, i.e. expect more desirable events and less undesirable events for the student character compared to the out-group characters [88, 89].

We first show that, within each character, participants expected desirable and undesirable events to occur differentially as a function of warmth and competence, even after balancing target events on key characteristics such as frequency, controllability, emotional intensity and personal experience. Respondents forecasted desirable events to occur more often than undesirable events for the student character and the elderly character but not for the businessperson or the alcoholic character. The expectation for more likely desirable events than undesirable events was strongest for the in-group member followed by the warm-but-less-competent out-group member. However, the competent-but-cold out-group member was expected to experience desirable and undesirable events with equal chances. The extreme out-group member (i.e. the univalent out-group member, low on both competence and warmth) was uniquely rated as having more chances of experiencing undesirable events than desirable events, mimicking the expectation for the in-group member but in the opposite direction. This pattern of findings mirrors the literature on desirability bias, which has shown that the respondents’ desires and motives influence how they generate likelihood estimates for future events [42, 58, 59]. The affective reactions to potential future events create motivational tendencies in respondents to approach desirable scenarios and avoid undesirable ones. These motivational tendencies, in turn, result in higher likelihood judgments for desirable events and lower judgments for undesirable events. In other words, respondents’ desires have a causal influence on the deliberation process. Desirability bias has been demonstrated for oneself [62, 116] and in-group members [17, 18, 38, 72, 74, 76]. Respondents are assumed to desire seeing themselves and close others in more positive situations than negative situations, inadvertently overestimating the likelihood estimates of the former and underestimating the latter. We can only speculate about the underlying cause of this desire. At face value, imaging and assessing the student character experiencing positive situations can be a reward in itself while imaging them being low on the risk of a negative situation reduces anxiety [58]. Another possibility is that the participants „need to believe that they live in a world where people generally get what they deserve”([117]; p. 1030–1031). This belief in a just-world prompts participants to expect more positive things for the deserving in-group student character and less positive things for to the undeserving extreme out-group member.

With the current results, we show that the desirability bias also operates on certain out-group members, albeit at a significantly reduced magnitude. The respondents expected more desirable than undesirable events for the warm mixed out-group member (i.e. the elder) but not the cold mixed out-group member (i.e. the businessperson). This is in line with the prediction that the warmth dimension holds more weight than the competence dimension in social perception [102, 118]. In the milieu of mixed emotions, the limited positive affect stemming from stereotypes of warmth seems sufficient to make respondents expect more desirable events than undesirable events happening to mixed warm out-groups. Contrary to the mixed warm character, the univalent out-group member triggers coherent negative emotions and attitudes [39, 90], which might be why our respondents pervasively expected more undesirable events than desirable events for this out-group.

In summary, we show that a specific form of optimistic bias was present in our respondents, which we term *desirability bias*, in line with previous research [13, 58, 59]. Respondents are invested in seeing the in-group member experience more desirable events than undesirable events but are motivated in seeing the univalent out-group member experience more undesirable events than desirable ones. These motivations are accurately predicted by stereotypes of warmth and competence, and they influence respondents’ likelihood estimates accordingly.

Our results also suggest that, within desirable and undesirable events, and across characters, stereotypes of warmth and competence consistently bias respondents’ likelihood estimates for out-group members, whereas likelihood judgments for oneself and the in-group member are influenced by the respondents’ personal experience with events. The hypotheses of comparative optimism in line with stereotypes of warmth and competence were supported for the three out-group characters. The univalent out-group member was rated with the least chances of experiencing desirable events and the highest chances of going through undesirable situations. The mixed out-group members were rated intermediately between the univalent out-group member and the in-group member. However, the predictions of comparative optimism regarding the in-group member’s likelihood estimates were only partially confirmed. Our student participants expected the fellow student character to have the highest chances of experiencing desirable events, as predicted. For undesirable situations, however, and contrary to predictions, participants also expected the fellow student character to have significantly high chances of experiencing them (i.e. higher than the two mixed out-group members and equal to the univalent out-group member). According to the SCM, the in-group member should be perceived as capable of not only maximizing positive outcomes but also minimizing negative outcomes by taking necessary preventive measures (i.e. stereotypes of competence; [89, 91]). Additionally, stereotypes of high warmth should elicit behaviors of active help and support from others, which should lead to further increased chances of desirable situations and decreased chances of undesirable outcomes, via indirect intervention of others[100, 101].

At face value, the unconfirmed predictions for the in-group member during undesirable events (as contrasted with the supported predictions for desirable events) could be interpreted as selective comparative pessimism. Although the literature points to an overall comparative optimistic bias among respondents, cases of comparative pessimism have been documented and several explanations have been put forth [25, 27, 28, 32, 59, 68, 119, 120]. One frequent factor that generates pessimism has been an undesirable event’s perceived severity, such that highly severe events can lead to particularly high likelihood estimates. Event severity has been operationalized inconsistently in the literature, either as high emotional impact of the undesirable target event [25, 59, 119] or, more often, as a lack of control over the outcome or occurrence of the undesirable situation [27, 28, 32, 44, 51]. In our pool of undesirable events, we sampled both controllable and less controllable events. Therefore, whatever pessimism might have been triggered by events low in control should have been balanced out by the events high in controllability. The aspect of high emotional impact of the undesirable events can equally be ruled out by looking at the scores of emotional intensity for undesirable events. Although desirable and undesirable events had overall similar scores of emotional intensity, the variability was even higher in the sample of undesirable events (a range of scores between 27 and 97 for undesirable events versus 44 and 79 for desirable events). Therefore, undesirable events lower in emotional intensity should have balanced out the events much higher in intensity. Other factors that can lead to comparative pessimism are high frequency of target events [36, 66, 68, 121] and increased personal experience with undesirable events [43, 120]. Although our sample of events included a balanced mix of rare and frequent events, the ratings of personal experience and perceived frequency highly correlated with each other, and with the likelihood estimates for oneself and for the in-group character. In addition, personal experience significantly predicted likelihood estimates for oneself and the in-group character but for none of the out-group characters. This suggests that it might not be the right end of the spectrum (i.e. only highly frequent events and the respondent’s high experience with the target events) that leads to increased likelihood estimates, but rather that likelihood estimates are a one-on-one mapping of the respondents’ experience with the events, regardless of whether the experience is high or low. This perspective can also easily explain the high likelihood estimates for desirable events, which, at face value, might be interpreted as selective comparative optimism.

Instead of explaining the results for the in-group member as selective comparative optimism and comparative pessimism, depending on the event’s valence, we suggest that other mechanisms may play a role in this pattern of findings. Specifically, the effects of event frequency and personal experience on likelihood estimates for oneself and in-group members can be accounted for by cognitive/egocentric biases such as differential sources of information, and differential amounts and accessibility to information [20, 58, 68, 122, 123]. The concepts of *differential amounts of information* and *differential accessibility of information* are interrelated and refer to the fact that respondents simply have more information (quantitatively or qualitatively) about themselves and others that they know directly, such as in-group members, and that they can access this information more readily than information about lesser-known others such as out-group members. The account of *differential sources of information* further extends this assumption by claiming that respondents use different types of knowledge when they think about themselves and close others, and when they think about lesser-known others. People rely on episodic memories to simulate future situations involving themselves and close others [124–127] and on idiosyncratic attributes and qualities to evaluate themselves and in-group members [128]. However, they rely on stereotypical knowledge and superficial processing to evaluate out-group members [129, 130]. The different types of sources of information for close and distant others could reflect differential processing of self and others at a neural level [131, 132].

In summary, we argue that cognitive/egocentric biases such as differential sources of information, differential amounts and access to information, can more readily explain the collective pattern of results for the in-group member for both desirable events and undesirable events. The fact that personal experience and event frequency significantly predicted the likelihood estimates for the in-group member but for none of the out-group members implies that respondents base their estimates for the latter on other factors. One such factor is the event’s perceived controllability. Likelihood estimates for the mixed character of the businessperson and the univalent out-group character were both correlated with and were predicted by the event’s controllability. For example, likelihood estimates for the univalent out-group member increased for undesirable events as the event’s perceived controllability also increased. For desirable events, likelihood estimates decreased as the perceived controllability increased. One might ask how the controllability of an event could influence likelihood estimates for out-group members, in particular. As argued above, respondents use stereotypical knowledge to assess future situations for out-group members [129, 130] and the content of this stereotypical knowledge is dictated by dimensions of warmth and competence [100, 108]. The link between event controllability, on one hand, and warmth and competence, on the other hand, can reasonably be made with Heckhausen’s theory of control [133], according to which people can exert control over life situations through direct means (i.e. primary control) and indirect means (i.e. secondary control). Primary control is akin to the SCM dimension of competence, whereas secondary control is akin to the warmth dimension [134]. Because primary control involves direct action taken by individuals to change outcomes or situations, it readily suits stereotypes of competence, such as intelligence, skill and efficiency [103, 111]. Secondary control refers to indirect ways of influencing a situation such as vicarious control, e.g. relying on competent others to help them maximize positive gains and prevent negative consequences, and thus readily accommodates the behaviors triggered by the warmth dimension [134]. An event’s perceived controllability could therefore influence likelihood estimates for out-group members via a combination of stereotypes of competence and warmth, which are proxies for primary and secondary control. Individuals perceived as low on competence lack the capability of directly controlling event outcomes, whereas individuals low on warmth trigger neglecting or even harmful behaviors from individuals or institutions who, if in a position of competence, could act to the referent’s detriment [50, 89, 104, 109].

The lack of definitive proof for comparative optimism for the in-group member in relation to out-group members adds to the recently voiced concerns about the true prevalence of comparative optimism [12, 66, 68]. A sample of target events unbalanced on either valence, emotional intensity, frequency, controllability or personal experience can bring about spurious effects of comparative optimism. In this regard, numerous studies that focused on different key event characteristics at a time have revealed inconsistent results [21, 27, 30, 31, 36, 45, 62, 110, 121, 135], while attempts at replicability have been largely unsuccessful [30, 31, 61, 136]. Nevertheless, the emergence of a strong desirability bias in our sample of respondents showcases overoptimistic expectancies and emphasizes the role of motivational forces in developing them [41, 42, 58]. Specifically, respondents forecasted that the in-group character would experience significantly more desirable events than undesirable events, despite the two types of events being similar in terms of controllability, frequency, emotional intensity and personal experience. This causal influence of the event’s valence on likelihood estimates was mirrored in the case of the extreme out-group member, whose chances of experiencing undesirable events significantly surpassed the chances of desirable events. Concomitantly, cognitive/egocentric biases also played a significant role, with respondents tapping into differences sources of information when evaluating the self and the in-group member (i.e. personal experience with the target events) and when evaluating out-group members (i.e. stereotypes of warmth and competence). The co-occurrence of motivational and cognitive/egocentric biases in likelihood estimates is in line with previous reports and reviews and suggests that not one type prevails over the other [15, 42, 58, 63, 122].

### Strengths and limitations

Our study presents several strengths and some limitations. First, we successfully addressed several methodological confounds that have been recently voiced [12, 20, 67, 68] by balancing desirable and undesirable events on a series of key event characteristics that have each been shown to moderate the extent of the optimistic bias, namely perceived frequency and controllability of events, emotional intensity and personal experience [21, 37]. Another strength of our study is that we successfully conceptualized the reference target in terms of warmth and competence, which are more adequate concepts than previously investigated characteristics such as social distance and level of similarity. Past research has conceptualized these characteristics inconsistently, either as social distance [24], vocational similarity [1], self-generated similarity [28], the amount of information known about someone [69] or left entirely to the understanding of the participant [11, 26, 29, 30, 51]. The SCM uses the warmth and competence dimensions to make predictions about intergroup emotions, attitudes and behaviors [89, 137].

Despite these strengths, we acknowledge some limitations of our experimental manipulations. The manipulation check of the SCM characters revealed a significant degree of entanglement between the dimensions of warmth and competence. The two-dimensional space provided by warmth and competence should ideally be inhabited by four stereotypes that differ *between* dimensions but not *within* dimensions. For example, the two characters designated as warm should differ in overall scores of perceived warmth from the two characters designated as cold, but they should not differ from one another. In our sample, the manipulation check revealed that the four characters differed *between* dimensions, as expected, with main effects of warmth and competence. However, there were also significant differences *within* dimensions. Specifically, the two warm characters (student and elder) differed in their levels of (high) warmth, the two competent characters (student and businessperson) differed in their levels of (high) competence, whereas the two less competent characters (elder and alcoholic) differed in their levels of (low) competence. As a result, throughout the manuscript we used the terms “high” and “low” in relation to the dimensions of competence or warmth in a relative manner rather than absolute. For instance, the student character, designated as high on warmth and high on competence, was, in fact, rated second highest in warmth (after the elder) and second highest in competence (after the businessperson). As such, the student character was considered “high” in warmth relative to the businessperson and the alcoholic, and “high” in competence relative to the elder and the alcoholic.

One likely factor that might have contributed to the *within dimension* differences is our use of direct questions to capture the perceived warmth and competence of the characters. In the SCM research, it is customary that questions assessing warmth and competence are asked indirectly through proxy constructs such as trustworthiness and intelligence, respectively, and an aggregate score is then computed for each SCM dimension [103, 137]. To maintain our experiment length to a minimum, we opted for two direct questions about the perceived warmth and competence, which might have skewed the results in at least two ways. First, a single question about the warmth and competence dimensions of the four characters may have led to a reduced capture of the complexity of the two dimensions. Prior studies have used up to six attributes per dimension to capture the different facets of warmth and competence, respectively [90, 106, 138, 139]. Second, prompting direct answers about particularly sensitive items such as social stereotypes might readily trigger a social desirability bias in participants [140]. The most problematic character in terms of within and between dimension differences was the elderly character. However, the mixed stereotype of low competence and high warmth pertaining to elderly people has previously been validated using indirect questions in several cross-national samples from Europe, Americas, Asia, Oceania and Africa [94, 109, 112]. We are, therefore, confident that the surprising results of the manipulation check for the elderly character, particularly the unusually high competence ratings, are likely an artefact resulting from a different methodology of collecting data. Furthermore, because the manipulation check for the elderly character could still reflect genuine evaluations of warmth and competence, we performed several additional analyses on a subset of the data to control for the *within* dimension differences. Specifically, we performed four ANCOVAs on the designated target dimension (e.g. competence) while controlling for differences in the non-target dimension (e.g. warmth). These results confirmed the initial three-way ANOVA, in that the warmth- and competence-informed hypotheses strongly hold for the three out-group members but not the in-group member.

Another limitation pertaining to our experimental manipulation is that the four fictional characters may also differ on dimensions other than warmth and competence. Future studies could match the characters more closely on as many dimensions as possible. We note, however, that a perfect match would be unreasonable to expect. Because our sample consisted predominantly of women, we cannot make strong generalizations about our findings. Future studies could balance the gender distribution and include gender and a between-subjects factor in the analyses.

Lastly, we acknowledge that the lack of neutral situations in our study limited our conclusions about the magnitude of motivational and cognitive/egocentric biases that respondents engage in, such as the desirability bias and differential sources of information, respectively. A much stronger case could be made for the effects of cognitive/egocentric biases if the respondents evaluate the self and in-group members in neutral events also as a function of their personal experience with the target events, as they did with desirable and undesirable events. Similarly, the case for a desirability bias could be further strengthened if respondents evaluate desirable events to occur more often than neutral events, and undesirable events less often than neutral events. Thus, future studies can include neutral events along with desirable and undesirable events, equally balanced on key characteristics.

## Conclusions and future directions

In conclusion, we showed that respondents manifest a particular type of optimistic bias, which we refer to as desirability bias, even after controlling for key event characteristics such as frequency, controllability, emotional intensity and personal experience. The magnitude and the direction of the desirability bias for the four fictional characters were contingent on stereotypes of warmth and competence, two universal dimensions of social perception. Specifically, participants expected that the in-group member and the two mixed out-group members would experience significantly more positive events than negative events, all other things being equal. The magnitude of this desirability bias was highest for the in-group member, followed by the warm-but-not-competent out-group member and, lastly, by the cold-but-competent out-group member. For the univalent negative out-group, the desirability bias manifested in the opposite direction, with participants expecting significantly more negative events than positive. The finding of a desirability bias for the in-group member suggests motivational forces to approach desirable events and avoid negative events, which, in turn, lead to overestimation and underestimation of likelihood estimates, respectively [13, 42, 58, 62]. The presence of a desirability bias in the opposite direction for the univalent out-group members suggests the malicious pleasure of the out-group’s misfortune (i.e. Schadenfreude [141, 142]). We further showed that, within desirable and undesirable events, respondents relied on different types of information sources when estimating the chances of experiencing identical events for oneself and the in-group member, on one hand, and for out-group members, on the other hand. Respondents tended to anchor the judgments for oneself and in-group member on their personal experience with target events but turned to stereotypical knowledge to judge out-group members. The differential sources of information, as well as differential amounts and accessibility to information relate to distinct cognitive/egocentric biases when evaluating in-group and out-group members.

These results have several implications for both optimism research and SCM research. First, our findings raise the possibility that comparative optimism for the self and in-group members is not ubiquitous and that a narrower definition of optimism may be more appropriate (i.e. as desirability bias: expecting more desirable events than undesirable events). Future studies should, foremost, include in their analyses both desirable and undesirable events, balanced on key event characteristics. Second, both motivational biases (i.e. expecting more desirable events to happen than undesirable events) and cognitive/egocentric biases (i.e. differential sources of information, differential amount and accessibility to information) influence different facets of likelihood estimates. Researchers should thus consider both accounts when designing their experiments and interpreting their findings. Third, the dimensions of warmth and competence could help explain likelihood estimates when the comparison target is an out-group member. Past research on overoptimistic beliefs has focused on “the average other” as a comparison target and has quantified the optimistic bias for oneself as a function of social closeness and similarity to the comparison target [1, 11, 23, 24, 26–30, 37, 51, 69]. Because warmth and competence incorporate these previously studied characteristics but offer a clearer conceptualization and higher predictive power [39, 88], we argue that our results expand on the past studies. As such, one can better interpret the „average other” from previous research as a comparison target of unknown warmth and competence. The previously found downward comparisons between a respondent and the reference target in comparative optimism [11, 143] may not be the direct result of the target being too abstract or not concrete enough per se, but rather the result of the target possessing unknown levels of competence and warmth. It has been shown that during intergroup comparisons with concrete out-groups, individuals freely choose traits on the two-dimensional space of warmth and competence that are favorable for themselves and their in-group but detrimental for the out-group [88–93]. The reverse scenario could be equally plausible: given an unknown comparison target (i.e. “the average student”), respondents choose a target whose levels of warmth and competence fit the trait dimensions that would be most favorable for themselves and detrimental for the comparison target. That is because, when asking respondents to answer specific comparative questions (i.e. requiring a comparison with a reference target on a given attribute), the researcher is implicitly providing the respondents with ready-made traits and respondents are prone to a self-enhancement bias.

Regarding the Stereotype Content Model, our results showed that stereotypes of warmth and competence more readily explain estimate likelihoods for out-group members than for oneself and the in-group member. The mismatch between the SCM predictions and our findings for the in-group member could be due to at least two reasons. First, it is possible that the cognitive/egocentric biases that drive the evaluations of the in-group member are strong enough to override the warmth- and competence-informed predictions of the SCM for the in-group member. Past research has singled out the potency of cognitive-egocentric biases when explaining the overoptimistic beliefs for oneself [13, 122] and in-group members [144]. Second, the SCM model might generally suffer from a lack of predictive power for in-group members. In fact, previous research has shown that the SCM can successfully forecast how respondents perceive and evaluate a variety of out-groups [90, 92, 137] but less so for in-group members [145, 146]. We, therefore, encourage future research into SCM to more amply test warmth- and competence-informed predictions about in-group members.

We also suggest potential lines of future research. For example, one can use an Implicit Association Test to measure the degree to which the four fictional characters are automatically associated with positive and negative concepts. As a manipulation check of the stereotypes associated with the fictional characters, this test can be implemented at the end of the main task. One can also experimentally reduce the implicit prejudice associated with the four social characters and investigate any ensuing changes in the likelihood estimates. A reduction in implicit prejudice can be prompted by a brief direct contact with a stereotyped person, e.g. elderly person or alcoholic, in an ecologically valid real-life interaction with that person (e.g. [147]). Alternatively, one can investigate empathy as a predictor of likelihood estimates depending on the reference. For example, the SCM posits that cognitive appraisals of structural relationships (status and competition) predict cognitive beliefs about social groups (stereotypes of warmth and competence). The latter can lead to behavioral tendencies towards social groups (i.e. help or harm) directly or indirectly ((via triggered emotions, e.g. pride, pity, envy, and disgust); [89]). Emotional and cognitive empathy can influence likelihood estimates by modulating stereotypes of warmth and competence at various levels, e.g. the cognitive appraisals of structural relationships, the formed beliefs of warmth and competence or the felt emotions towards social groups.

## Supporting information

S1 Analysis Manipulation check of the events. (DOCX)

S2 Analysis Manipulation check of the characters. (DOCX)

S1 Discussion Discussion of the manipulation check of characters. (DOCX)

S1 Fig Pirate plots of the perceived warmth scores for each character. (PNG)

S2 Fig Mapping of the four characters on a two-dimensional space of perceived warmth and perceived competence. (PNG)

S3 Fig Pirate plots of the perceived competence scores for each character. (PNG)

S4 Fig Example of an identical event (“Being hugged”) depicting each of the four characters. (PNG)

S5 Fig Layout of a trial from the SCM-based experimental task with the character as the reference target. (PNG)

S6 Fig Layout of a trial from the SCM-based experimental task with the self as the reference target. (PNG)

S7 Fig Example of instructions from the Inclusion of Other in the Self task. (PNG)

S1 Table The exhaustive list of events used in the experiment. (DOCX)

S2 Table Event characteristics along with their mean and standard deviation. (DOCX)

S3 Table Bivariate correlations between the five event characteristics. (DOCX)

S1 Data File Raw data of all described analyses. (XLSX)
