# Supplementary material for: Warmth and competence predict overoptimistic beliefs for out-group but not in-group members
Source: PLoS One. 2018 Nov 26;13(11):e0207670. doi: 10.1371/journal.pone.0207670 (PMC6261057; doi:10.1371/journal.pone.0207670)
Supplement: S1 Analysis — (DOCX) [file pone.0207670.s001.docx]

## S1 Analysis. Manipulation check of the events.

The success of our assessment task depended on life scenarios with a balanced range of perceived frequency and controllability in the general population, as well as perceived emotional intensity, desirability, and personal experience with the events. The designated desirable and undesirable events were perfectly balanced, with all undesirable events averaging below 50 (*M* = 21.31, SD = 12.23) and all desirable events averaging over 50 (*M* = 79.36, SD = 10.97; **S2 Table**). With an adjusted alpha level of 0.01 (Bonferroni family-wise error rate correction for five comparisons), paired t-tests revealed a statistically significant difference in scores of valence (t (7) = 16.77, p < .0005, r = 0.97) between desirable (*M* = 79.36, SD = 4.82) and undesirable events (*M* = 21.31, SD = 13.11) but no statistically significant difference in scores of emotional intensity (t (7) = .684, p = .504, r = .17). Furthermore, there were no statistically significant differences in scores of perceived frequency (t (15) = .994, p =.336, r = .25), perceived controllability (t (15) = .556, p = .587, r = .14) or personal experience (t (15) = 2.547, p = .022, r = .55) between desirable and undesirable events.

In addition, we performed correlations between the five event characteristics to determine whether participants rated the events on one characteristic by extrapolating the other characteristics. For both desirable and undesirable events, scores of perceived valence significantly correlated with scores of emotional intensity (desirable events: r = .56, p = .024; undesirable events: r = -.88, p < .0005), suggesting that participants rated the emotional intensity of the event based on how positive or negative the event was perceived (i.e. how further away the event’s valence was from a hypothetical middle point, implying an emotionally neutral event). Specifically, they rated an undesirable event as more emotionally intense as the perceived valence of the event was closer to the most leftward value of the emotional valence scale. They rated a desirable event as more emotionally intense as the perceived valence of that event was closer to the most rightward value of the emotional valence scale. In addition, scores of perceived frequency significantly correlated with scores of personal experience (desirable events: r = .95, p <.0005; undesirable events: r = .88, p < .0005), strongly suggesting that participants gauged the event’s frequency in the general population based on their own experience with the target event. Specifically, as personal experience with the target event increased, so did the hypothesized incidence of that event in the general population. For undesirable events, scores of frequency additionally correlated moderately with valence (r = .55, p =.026), emotional intensity (r = -.56, p = .024) and controllability (r = .69, p = .003), while scores of personal experience correlated with valence (r = .65, p = .006), emotional intensity (r = -.70, p = .003) and controllability (r = .54, p = .030). **S3 Table** shows all correlation coefficients between the five event characteristics and the corresponding significance levels.

In summary, the results presented above suggest that the designated desirable and undesirable events were matched on controllability, frequency, emotional intensity, and personal experience.
