# Supplementary material for: Warmth and competence predict overoptimistic beliefs for out-group but not in-group members
Source: PLoS One. 2018 Nov 26;13(11):e0207670. doi: 10.1371/journal.pone.0207670 (PMC6261057; doi:10.1371/journal.pone.0207670)
Supplement: S2 Analysis — (DOCX) [file pone.0207670.s002.docx]

## S2 Analysis. Manipulation check of the SCM characters.

To determine whether the participants identified with the student characters as hypothesized and, thus, whether the in-group-out-group manipulation worked, we analyzed the ratings in the IOS task. A one-way repeated measures ANOVA with the factor **character** (four levels: alcoholic, elderly, businessperson, student) on scores of the Inclusion of Other in the Self measure revealed significant differences in how much participants identified with each character (F (2.43, 213.85) = 229.86, Greenhouse-Geisser correction, p < .0005). Pairwise comparisons using the Bonferroni correction revealed that all characters were rated significantly different from each other (all *p*s < .0005): the participants identified the most with the student character (*M* = 6.03, SD = 1.31), followed by the elderly character (*M* = 3.53, SD = 1.67), the business person (*M* = 2.34, SD = 1.15) and the alcoholic character (*M* = 1.51, SD = 0.83.

To provide a quick post-hoc check on whether the assumptions of designated warmth and competence would hold in our sample, we analyzed the scores of perceived warmth and competence of each of the four characters. Repeated measures ANOVA with the factor **character** (four levels: student, elderly, businessperson, alcoholic) on scores of perceived *warmth* showed a statistically significant difference between characters (F (3,264) = 380.35, Greenhouse-Geisser correction, p < .0005, *η*_p_^2^ = .814). Post-hoc tests using the Bonferroni correction revealed that the elderly character (*M* = 88.35, SD =11.78) was rated significantly warmer than the student character (*M* = 75.48, SD = 11.60; p < .0005), the businessperson (*M* = 30.58, SD = 17.30, p < .0005) and the alcoholic character (*M* = 28.07, SD = 17.30, p < .0005; **S1 and S2 Figs**). The student character was also rated significantly warmer than the businessperson (p < .0005) and the alcoholic character (p < .0005). However, the businessperson and the alcoholic characters were rated as similarly cold (p = 1.00). Repeated measures ANOVA with the factor **character** (four levels: student, elderly, businessperson, alcoholic) on scores of perceived *competence* showed a significant difference between characters (F (3,261) = 389.10, sphericity assumed, p < .0001, *η*_p_^2^ = .817). Post-hoc tests with the Bonferonni correction revealed that all characters differed in perceived competence (p < .0005): the businessperson (M = 87.36, SD = 13.41) was rated the highest, followed by the student character (*M* = 71.80, SD = 15.14), the elderly character (*M* = 57.10, SD = 17.49) and the alcoholic character (*M* = 13.91, SD = 14.40, **S2 and S3 Figs**).
