# Supplementary material for: Warmth and competence predict overoptimistic beliefs for out-group but not in-group members
Source: PLoS One. 2018 Nov 26;13(11):e0207670. doi: 10.1371/journal.pone.0207670 (PMC6261057; doi:10.1371/journal.pone.0207670)
Supplement: S1 Discussion — (DOCX) [file pone.0207670.s003.docx]

**S3 Discussion. Discussion of the manipulation check of characters.**

Because the post-hoc manipulation check revealed that some of the four fictional characters differed on more than one dimension, it was possible that the findings pertaining to the designated target dimension (e.g. competence) could be confounded by differences in the perceived non-target dimension (e.g. warmth). We thus ran several additional analyses to separately test for the effects of the designated target dimensions of warmth and competence on the likelihood estimates, each time controlling for differences in the other dimension. Specifically, we first ran two two-way repeated measures ANCOVAs with factors competence (high versus low) and desirability (desirable versus undesirable events) on the likelihood estimates. For the factor competence in the first two-way ANCOVA, we looked at one prototypical pair of designated high competence versus low competence: the businessperson (i.e. high competence) and the alcoholic character (i.e. low competence). In the second two-way ANCOVA, the factor competence included the prototypical pair of the student character (i.e. high competence) and the elderly character (i.e. low in competence). For both ANCOVAs, we computed the differences in perceived warmth between the two respective characters in each pair and we included the values as a covariate. For the ANCOVA on the businessperson-alcoholic character, we found no main effect of designated competence (F (1,80) = .32, p = .571, η_p_^2^ = .004) but there was a main effect of desirability (F (1,80) = 54.50, p < .0005, η_p_^2^ = .405) which was qualified by an interaction effect (F (1,80) = 151.07, p < .0005, η_p_^2^ = .645). The participants rated the businessperson as having significantly more chances of experiencing desirable events (*M* = 50.96, SD = 10.18) than the alcoholic character (*M* = 38.59, SD = 12.87), thus supporting our hypothesis H1a. Compared to the businessperson (*M* = 48.17, SD = 11.08), participants expected that the alcoholic character will experience significantly more undesirable events (*M =* 59.01, SD = 10.38), thus supporting our hypothesis H1a. For the ANCOVA on the student character-elderly character, there was a main effect of competence (F (1,80) = 100.39, p < .0005, η_p_^2^ = .557) and valence (F (1,80) = 212.64, p < .0005, η_p_^2^ = .727) but no interaction effect (F (1,80) = 1.77, p = .188, η_p_^2^ = .022). Participants expected desirable events (*M* = 66.01, SD = 10.12) to occur significantly more often than undesirable events (*M* = 52.65, SD = 10.79) for both the student and the elderly characters. At the same time, the student character was overall rated with significantly more chances of experiencing the events (*M =* 64.79, SD = 10.49) compared to the elderly character (*M* = 53.88, SD = 10.42). Overall, the pattern of results of the student-elderly pair does not provide support for our hypotheses. Instead, these ANCOVA findings echo the results of the previous three-way repeated measures ANOVA. Specifically, the participants expected the student (i.e. in-group member; high in competence) to experience not only more desirable events but also more undesirable events than the elder (i.e. low in competence).

Secondly, we ran two additional two-way repeated measures ANCOVA on the likelihood estimates with factors warmth (high versus low) and desirability (desirable versus undesirable events). For the factor warmth in the first ANCOVA, we looked at the prototypical pair of designated high warmth vs low warmth: the student character (i.e. high warmth) and the businessperson (i.e. low warmth). For the second ANCOVA, the prototypical comparison was between the elderly character (i.e. high warmth) and the alcoholic character (i.e. low warmth). For both ANCOVAs, we computed the differences in perceived competence between the two respective characters in each pair, and we included the values as a covariate. For the ANCOVA on the student versus businessperson, there was a main effect of warmth (F (1,80) = 117.89, p < .0005, η_p_^2^ = .596), with the student character being rated with significantly more chances of experiencing events, in general, than the businessperson. A main effect of desirability (F (1,80) = 53.02, p < .0005, η_p_^2^ = .399) was qualified by an interaction with warmth (F (1,80) = 28.81, p < .0005, η_p_^2^ = .265). The participants expected overall more desirable events (*M* = 60.87, SD = 10.08) than undesirable events (*M* = 53.47, SD = 11.04) and this difference was greater for the student character (*M* = 12.02) than for the businessperson (*M* = 2.77). These findings support our hypothesis H2a concerning desirable events and hypothesis H2b concerning undesirable events. For the ANCOVA on the elderly character versus alcoholic character, we found no statistically significant main effects of warmth (F (1,80 = 2.28, p = .135, η_p_^2^ = .028) or desirability (F (1,80) = 0.72, p = .789, η_p_^2^ = .001) but there was a significant two-way interaction effect (F (1,80) = 44.32, p < .0005, η_p_^2^ = .356). The participants expected desirable events to happen significantly more often to the elderly character (*M* = 61.24, SD = 10.28) than the alcoholic character (*M* = 38.59, SD = 12.87), and expected undesirable events to happen significantly more often to the alcoholic character (*M* = 59.01, SD = 10.38) than the elderly character (*M* = 38.59, SD = 12.87). These results support our hypothesis H2a concerning desirable events and hypothesis H2b concerning undesirable events.
