# Supplementary figures and images for: Warmth and competence predict overoptimistic beliefs for out-group but not in-group members
Source: PLoS One. 2018 Nov 26;13(11):e0207670. doi: 10.1371/journal.pone.0207670 (PMC6261057; doi:10.1371/journal.pone.0207670)

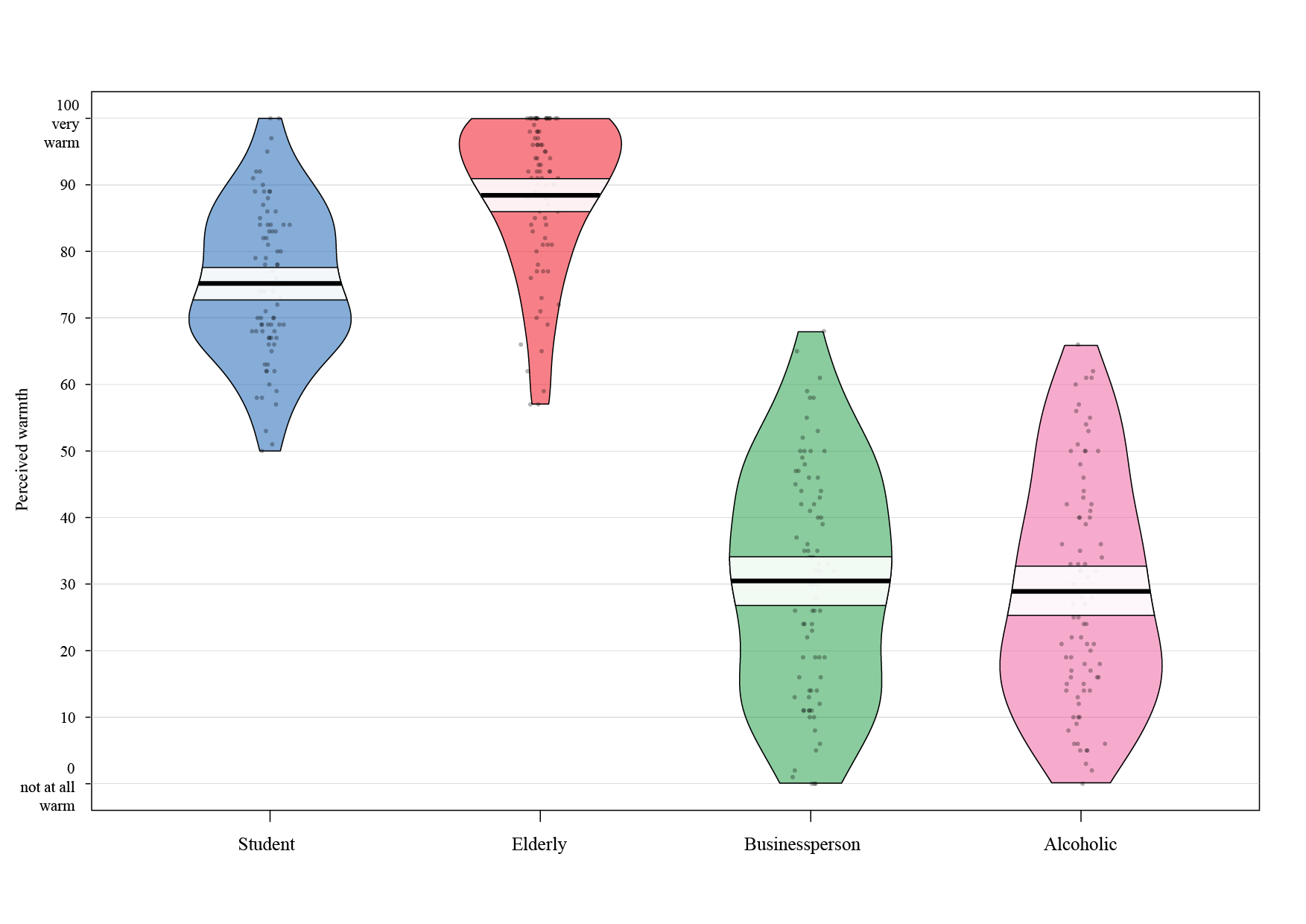

Supplement: S1 Fig — (PNG) [file pone.0207670.s004.png]

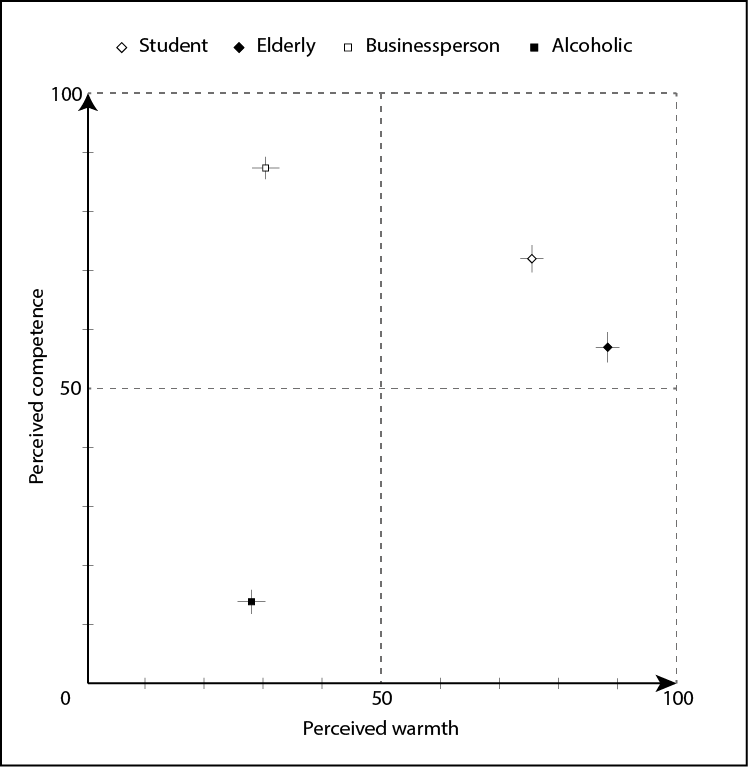

Supplement: S2 Fig — (PNG) [file pone.0207670.s005.png]

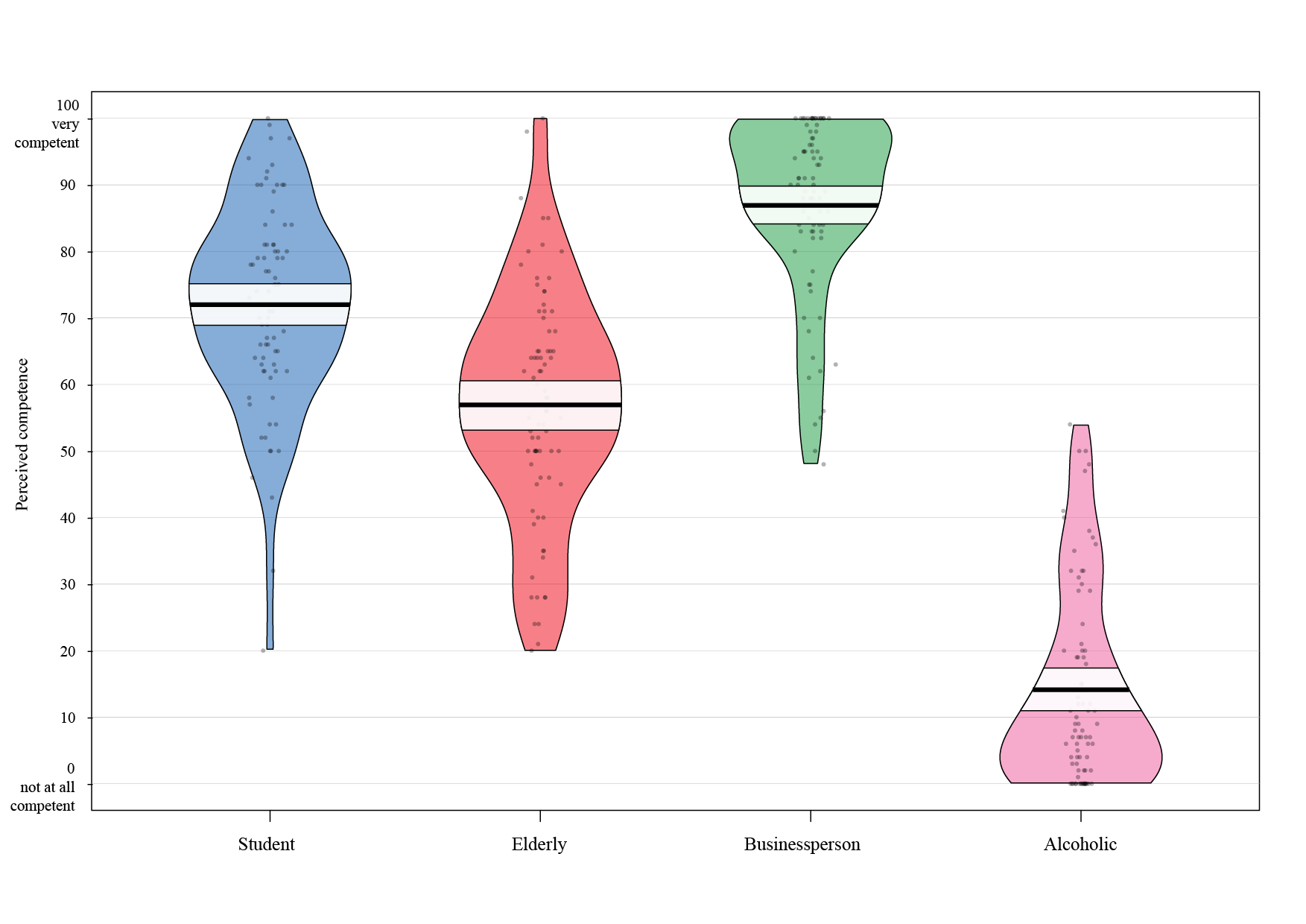

Supplement: S3 Fig — (PNG) [file pone.0207670.s006.png]

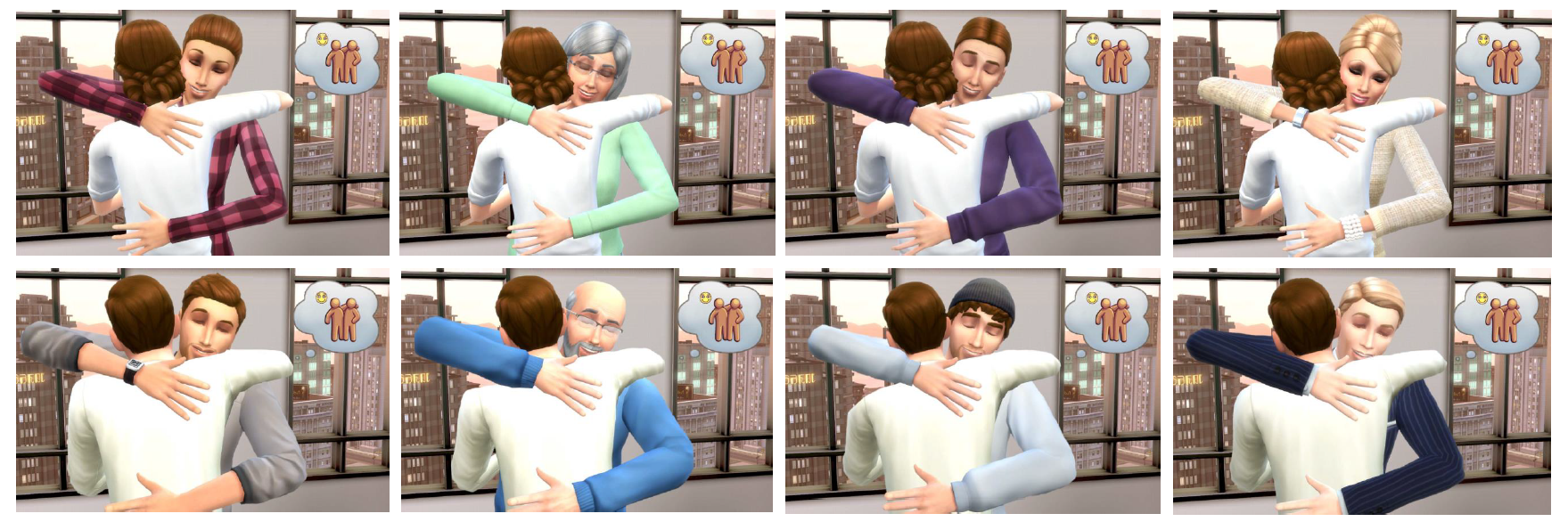

Supplement: S4 Fig — (PNG) [file pone.0207670.s007.png]

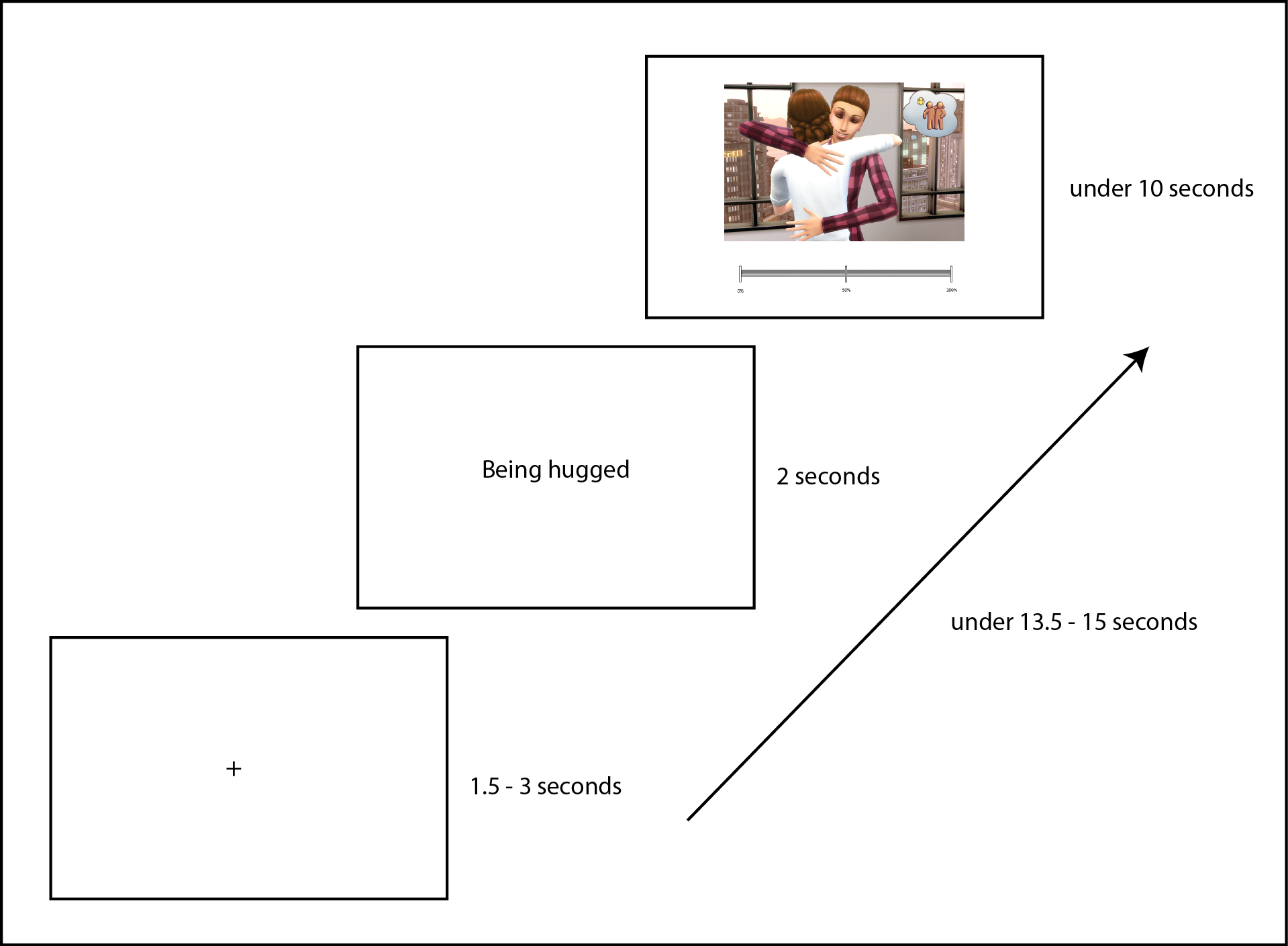

Supplement: S5 Fig — (PNG) [file pone.0207670.s008.png]

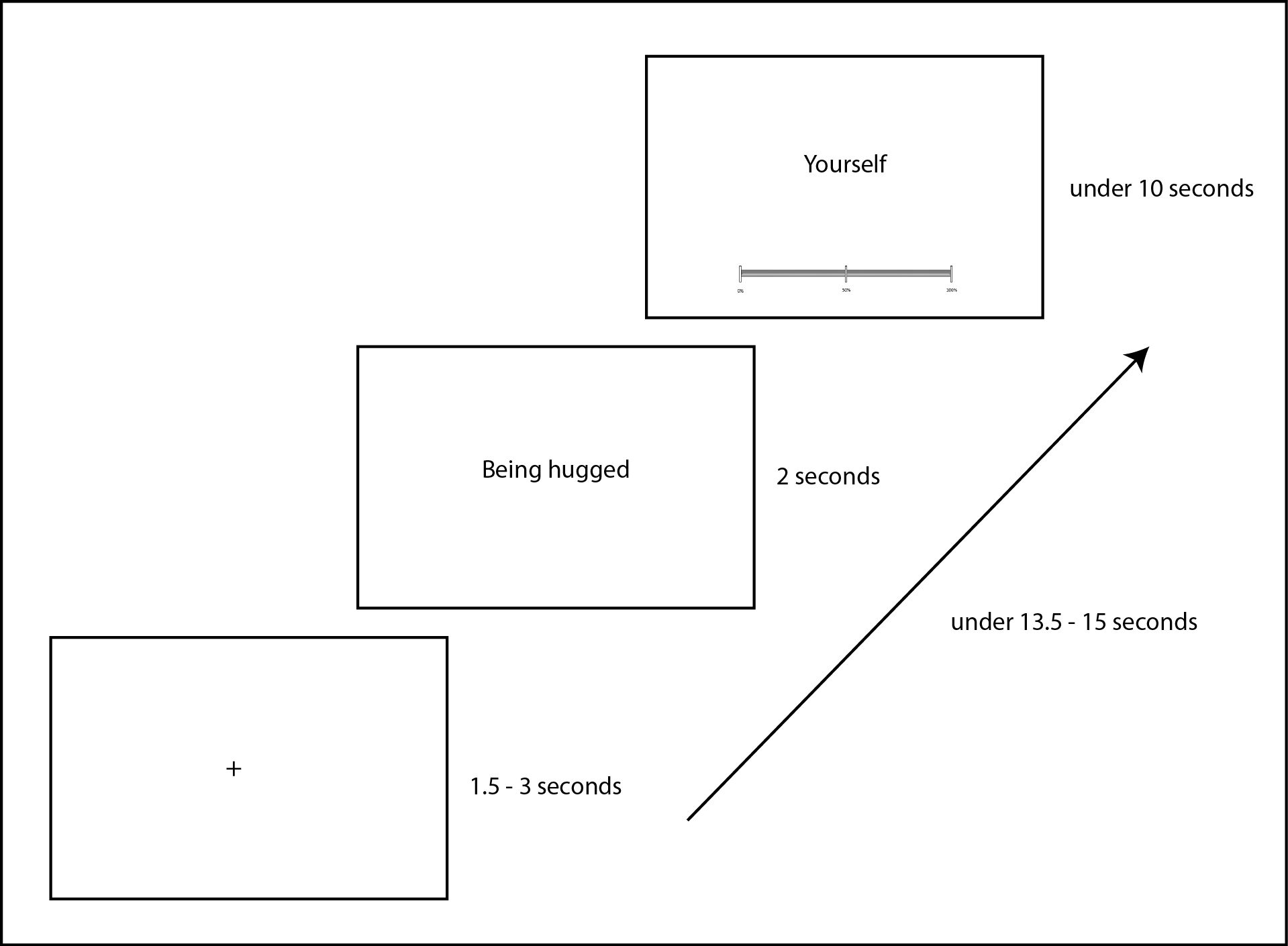

Supplement: S6 Fig — (PNG) [file pone.0207670.s009.png]

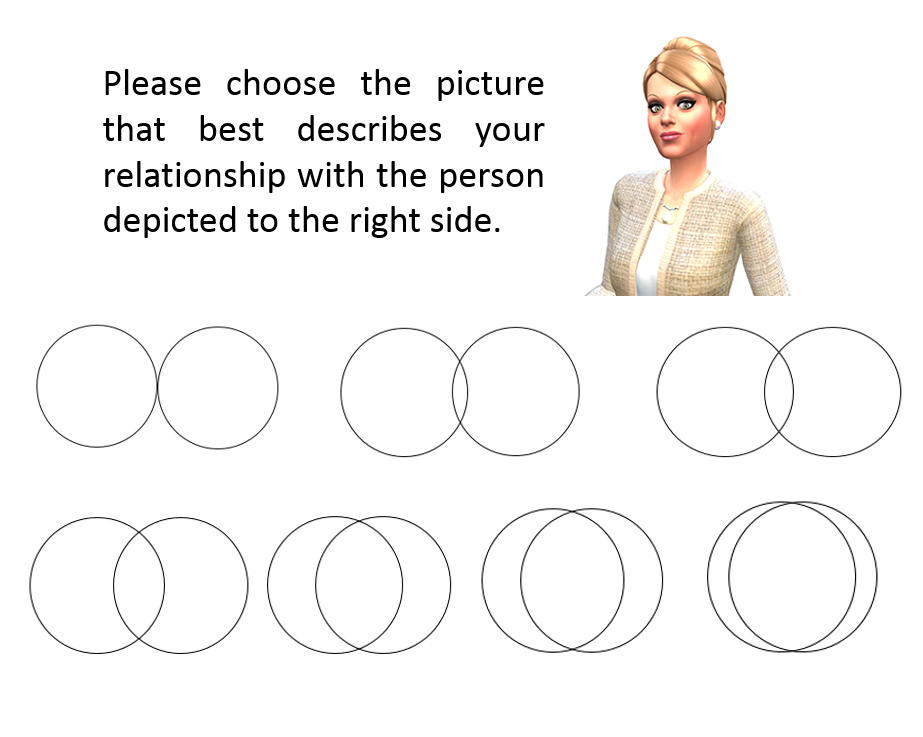

Supplement: S7 Fig — (PNG) [file pone.0207670.s010.png]
