# Supplementary material for: Warmth and competence predict overoptimistic beliefs for out-group but not in-group members
Source: PLoS One. 2018 Nov 26;13(11):e0207670. doi: 10.1371/journal.pone.0207670 (PMC6261057; doi:10.1371/journal.pone.0207670)
Supplement: S1 Table — (DOCX) [file pone.0207670.s011.docx]

**S1 Table. The exhaustive list of events used in the experiment.**

|  | Event | Desirability | Frequency | Controllability |
| --- | --- | --- | --- | --- |
| 1 | Being hugged ^a^ | High | High | High |
|  | A friend returns borrowed money to you | High | High | High |
|  | Being greeted warmly by relatives at a family celebration | High | High | High |
|  | Enjoy a warm bath or shower on a cold day | High | High | High |
| 2 | Hearing a very funny joke | High | High | Low |
|  | New neighbor comes over to introduce themselves | High | High | Low |
|  | A song that you like comes on the radio | High | High | Low |
|  | Bumping into an old friend on the street | High | High | Low |
| 3 | Delivering a speech successfully ^a^ | High | Low | High |
|  | A child has fun while you take care of him for 2 hours^a^ | High | Low | High |
|  | Winning a karaoke contest ^a^ | High | Low | High |
|  | Writing a bestseller about one’s own life ^a^ | High | Low | High |
| 4 | Win a car in the lottery | High | Low | Low |
|  | Find a 20 CHF bill on the ground | High | Low | Low |
|  | Seeing a comet in the sky | High | Low | Low |
|  | Win a sports bet ^b^ | High | Low | Low |
| 5 | The store closes just as you arrive | Low | High | High |
|  | Finding rotten food in the refrigerator | Low | High | High |
|  | Using a public restroom | Low | High | High |
|  | Drinking cold coffee/tee ^a^ | Low | High | High |
| 6 | A neighbor is listening to very loud music | Low | High | Low |
|  | Getting heartbroken after a relationship | Low | High | Low |
|  | Being confused with another person | Low | High | Low |
|  | Computer crashes in the middle of writing a text | Low | High | Low |
| 7 | Marriage ends in a bitter divorce | Low | Low | High |
|  | Lose 50 CHF ^a^ | Low | Low | High |
|  | Developing an excruciating toothache | Low | Low | High |
|  | Become a wound that needs to be sewn ^b^ | Low | Low | High |
| 8 | Witnessing a robbery of a bank | Low | Low | Low |
|  | Being falsely accused of a serious crime | Low | Low | Low |
|  | Receiving a dog bite | Low | Low | Low |
|  | Witnessing a tree falling on a house ^b^ | Low | Low | Low |

*Note*. Four examples for each possible combination of valence, frequency and controllability were used (Please refer to Table 1 for the possible combinations of event characteristics).

^a^ Newly created scenarios. ^b^ Scenarios rephrased and adapted from [1]

1. Chambers JR, Windschitl PD, Suls J. Egocentrism, event frequency, and comparative optimism: When what happens frequently is “more likely to happen to me”. Personality and Social Psychology Bulletin. 2003;29(11):1343-56.
