# Supplementary material for: Warmth and competence predict overoptimistic beliefs for out-group but not in-group members
Source: PLoS One. 2018 Nov 26;13(11):e0207670. doi: 10.1371/journal.pone.0207670 (PMC6261057; doi:10.1371/journal.pone.0207670)
Supplement: S2 Table — (DOCX) [file pone.0207670.s012.docx]

**S2 Table. Event characteristics along with their mean and standard deviation.**

|  | **Valence** | | **Emotional intensity** | | **Frequency** | | **Controllability** | | **Personal experience** | |
| --- | --- | --- | --- | --- | --- | --- | --- | --- | --- | --- |
|  | **Desirable** | **Undesirable** | **Desirable** | **Undesirable** | **Desirable** | **Undesirable** | **Desirable** | **Undesirable** | **Desirable** | **Undesirable** |
|  | 71.98 | 39.46 | 44.72 | 27.36 | 58.43 | 72.19 | 57.99 | 70.25 | 65.65 | 79.51 |
|  | 86.03 | 24.89 | 52.06 | 37.85 | 69.36 | 56.85 | 92.12 | 87.76 | 82.72 | 55.74 |
|  | 87.94 | 30.16 | 79.52 | 47.46 | 78.03 | 51.47 | 66.55 | 78.96 | 90.99 | 52.46 |
|  | 82.25 | 40.01 | 72.88 | 27.30 | 61.82 | 63.03 | 60.65 | 85.17 | 83.89 | 62.78 |
|  | 72.34 | 14.61 | 53.51 | 68.16 | 38.22 | 46.58 | 29.04 | 31.47 | 39.70 | 55.54 |
|  | 77.34 | 33.55 | 58.80 | 50.58 | 66.38 | 52.94 | 36.36 | 29.35 | 74.58 | 47.01 |
|  | 79.19 | 6.15 | 68.88 | 97.64 | 52.80 | 47.82 | 19.71 | 35.97 | 65.25 | 50.12 |
|  | 78.18 | 45.75 | 58.42 | 37.74 | 66.29 | 44.40 | 42.12 | 13.94 | 77.61 | 49.03 |
|  | 79.67 | 23.45 | 66.79 | 63.67 | 58.97 | 24.48 | 79.00 | 57.72 | 65.83 | 23.54 |
|  | 81.34 | 6.29 | 78.92 | 96.79 | 12.21 | 46.90 | 73.70 | 63.24 | 2.04 | 6.90 |
|  | 74.74 | 17.10 | 63.34 | 62.40 | 24.74 | 36.34 | 62.18 | 57.79 | 13.55 | 34.20 |
|  | 80.04 | 9.42 | 68.81 | 57.20 | 43.11 | 35.03 | 81.57 | 46.22 | 54.09 | 34.19 |
|  | 78.90 | 16.02 | 54.72 | 68.57 | 21.36 | 9.25 | 10.27 | 7.47 | 34.85 | 5.54 |
|  | 73.85 | 4.94 | 67.84 | 88.58 | 23.47 | 15.04 | 21.75 | 18.35 | 8.25 | 3.27 |
|  | 86.85 | 12.33 | 77.74 | 67.20 | 5.84 | 23.66 | 11.61 | 34.47 | 3.30 | 19.90 |
|  | 79.08 | 16.89 | 63.25 | 75.57 | 26.38 | 9.62 | 19.90 | 8.09 | 50.37 | 2.22 |
| ***M*** | **79.36** | **21.31** | **64.39** | **60.88** | **44.21** | **39.73** | **47.78** | **45.39** | **50.79** | **36.37** |
| **SD** | **4.82** | **13.11** | **10.22** | **22.21** | **22.72** | **18.90** | **27.15** | **27.03** | **30.43** | **23.87** |

*Note*. The table presents mean (***M***) and standard deviations (**SD**). Scores were obtained with the help of a visual analog scale framed between 0 and 100.
