# Supplementary material for: Warmth and competence predict overoptimistic beliefs for out-group but not in-group members
Source: PLoS One. 2018 Nov 26;13(11):e0207670. doi: 10.1371/journal.pone.0207670 (PMC6261057; doi:10.1371/journal.pone.0207670)
Supplement: S3 Table — (DOCX) [file pone.0207670.s013.docx]

**S3 Table. Bivariate correlations between the five event characteristics.**

| **Desirable events** | Valence | Intensity | Controllability | Frequency | Experience |
| --- | --- | --- | --- | --- | --- |
| Valence |  | .560 ^a^ | .258 | .147 | .241 |
| Intensity | .560 ^a^ |  | .070 | -.247 | -.253 |
| Controllability | .258 | .070 |  | .453 | .349 |
| Frequency | .147 | -.247 | .453 |  | .948 ^a^ |
| Experience | .241 | -.253 | .349 | .948 ^a^ |  |
| **Undesirable events** | Valence | Intensity | Controllability | Frequency | Experience |
| Valence |  | -.883 ^a^ | .307 | .555 ^a^ | .653 ^a^ |
| Intensity | -.883 ^a^ |  | -.474 | -.560 ^a^ | -.700 ^a^ |
| Controllability | .307 | -.474 |  | .690 ^a^ | .541 ^a^ |
| Frequency | .555 ^a^ | -.560 ^a^ | .690 ^a^ |  | .882 ^a^ |
| Experience | .653 ^a^ | -.700 ^a^ | .541 ^a^ | .882 ^a^ |  |

*Note.* The table presents Pearson correlation coefficients. ^a^ p < .05, for all other entire p > .05
